# Robust and localised control of a 10-spin qubit array in germanium

**DOI:** 10.1038/s41467-025-65577-3

**Published:** 2025-11-26

**Authors:** Valentin John, Cécile X. Yu, Barnaby van Straaten, Esteban A. Rodríguez-Mena, Mauricio Rodríguez, Stefan D. Oosterhout, Lucas E. A. Stehouwer, Giordano Scappucci, Maximilian Rimbach-Russ, Stefano Bosco, Francesco Borsoi, Yann-Michel Niquet, Menno Veldhorst

**Affiliations:** 1 QuTech and Kavli Institute of Nanoscience, Delft University of Technology, Delft, The Netherlands; 2 Université Grenoble Alpes, CEA, IRIG-MEM-L_Sim, Grenoble, France; 3 QuTech and Netherlands Organisation for Applied Scientific Research, Delft, The Netherlands

**Keywords:** Quantum information, Electronic devices, Quantum dots, Qubits

## Abstract

Quantum computers require the systematic operation of qubits with high fidelity. For holes in germanium, the spin-orbit interaction allows for electric, fast and high-fidelity qubit gates. However, the strong g-tensor anisotropy of holes in germanium and their sensitivity to the operational and environmental conditions challenge the operation of large qubit arrays. Here, we investigate a two-dimensional 10-spin qubit array with single-qubit gate fidelities above 99%, and obtain surprisingly uniform qubit properties. By tuning the hole occupation, we demonstrate control over the spin susceptibility, enabling fast plunger gate driving with Rabi frequencies consistently above 1.45 MHz/ (mV ⋅ T). Moreover, we probe the locality of electric dipole spin resonance and find that the configuration with three-hole occupancy driven by the associated quantum dot plunger gate reduces crosstalk, lowering it by an average factor of 2.5 to nearest neighbours, compared to single-hole plunger driving. Theoretical modelling points towards the pronounced anisotropy of *p*-like orbitals as the main mechanism with significant contributions through Coulomb interactions, giving directions for reproducible control of large qubit arrays.

## Abstract (web summary)

Hole spin qubits in germanium are well suited for fast, electrically driven gates with high fidelity, but scaling to large qubit arrays remains challenging. Here the authors demonstrate a 10-spin qubit array with gate fidelities exceeding 99%, revealing mechanisms for uniform and scalable qubit control.

## Introduction

Semiconductor spin qubits have seen significant progress over the last few years, with four-qubit and six-qubit quantum processors demonstrated across different platforms and encodings^1–4^. In pursuit of scaling beyond these systems, larger quantum dot (QD) arrays have been explored, showcasing charge tune-up in a 4 × 4 QD array using a crossbar architecture^5^, qubit characterization of a two-dimensional 10-QD array by coherent single spin shuttling^6^, and demonstration of a linear array comprising 12 qubits^7^.

Hole spin qubits in planar strained Ge/SiGe heterostructures emerged as a compelling platform that can offer all-electrical control, fast Rabi driving, long coherence times, and absence of valley degree of freedom^8–10^. However, the strong anisotropy of the g-tensor opens a narrow window around the in-plane magnetic field direction, containing sweet but also weak operation spots for electric dipole spin resonance (EDSR) and coherence times^11–14^. The magnetic field directions of these optimal and weak spots are hard to predict and sensitive to electrostatic and strain fluctuations^15,16^, which differ across quantum dots due to device-specific and cooldown-dependent potential landscapes. Beyond optimizing global qubit parameters, such as material growth properties or magnetic field orientation, qubit performance can also be tuned by adjusting local control parameters, such as dot occupation or drive gate. For holes in silicon, it was observed that the hole occupancy can have little effect on the g-tensor^17,18^. For electrons in silicon operated with a micromagnet, it was shown that higher charge occupancies can increase the Rabi driving^19^. This was speculated to result from an increased mobility of the qubit charge in the magnetic field gradient, though more recent theoretical work also considered the effect of spin-orbit interaction via the quadrupolar contribution^20^. This raises the question whether the hole occupancy can be used to improve EDSR driving, in particular in systems with strong spin-orbit interaction, and whether further optimization is possible by choosing the gate drive.

In this work, we investigate a two-dimensional 10-QD device hosting 10 hole-spin qubits, with two central qubits each connected to four different neighbors. We systematically evaluate the EDSR drive efficiency of each qubit for QDs occupied with one, three, and five holes, to assess how charge configuration influences driving mechanisms. We apply a magnetic field slightly out-of-plane just outside the narrow window containing sweet and weak spots. This analysis is extended across all 22 available gates in the device for each qubit, which gives us insights about the locality and crosstalk of EDSR driving. Additionally, to probe the variation in noise sensitivity in each configuration, we perform longitudinal spin-electric susceptibility (LSES) measurements by analyzing the changes in resonance frequency as a function of gate voltages under different charge configurations, which is closely related to the qubit coherence. Crucially, we find that it is possible to have systematic, efficient driving with limited crosstalk when operating with three-hole occupancy using the plunger gate.

## Results

### The two-dimensional 10-spin qubit array

**Fig. 1:**
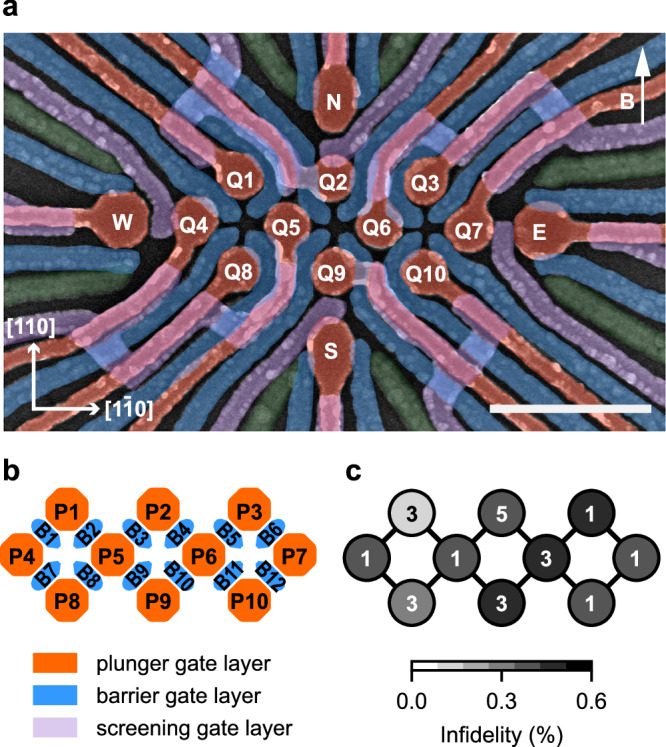
A high-fidelity 10-spin-qubit array in germanium. **a** A false-colored scanning electron microscope image of a nominally identical device, with the 10 quantum dot plunger gates highlighted in orange, the 12 barrier gates shown in blue, and the screening gates that screen the fanout of the plunger gates to avoid accumulation outside the quantum dot array in purple. Four single hole transistors labeled as N, E, W and S are located at the edge of the array. The 10 qubits are labeled as Q1–Q10. The applied magnetic field is 41.4 mT. The scale bar on the bottom right represents 500 nm. **b** Simplified gate layout of the quantum dot array where the plunger gates are labeled as P1–P10 and the barrier gates as B1–B12. **c** Randomized benchmarking single-qubit gate infidelities with the corresponding charge occupation of the 10 quantum dots annotated.

Figure 1a displays our device, comprising 10 QDs arranged in a 3-4-3 configuration, and four charge sensors located at the cardinal points of the array, identical to the device layout described in ref. ^21^. The fabrication details can be found in Supplementary Note 1. In this work, the quantum device is fabricated on a Ge/SiGe heterostructure grown on a germanium wafer^22^, exhibiting a high mobility of 3.4(1) × 10^6^ cm^2^/Vs, indicating a uniform and low-noise potential landscape for QD arrays^21^. The QDs are defined and operated using plunger and barrier gates, as illustrated in Fig. 1b. A magnetic field of 41.4 mT is applied by a uniaxial solenoid magnet, tilted approximately 2–3 degrees from the in-plane orientation^21^.

The 10-QD array is tuned to a dense charge configuration, with an odd number of holes at each QD site, defining 10 qubits labeled Q1–Q10. Each qubit is initialized and readout pairwise using Pauli spin blockade with a nearby charge sensor^23,24^. Figure 1c shows the occupation of each QD in the initial tune-up, along with the corresponding single-qubit gate infidelity, all below 0.6%, obtained through randomized benchmarking^25^ (see Supplementary Note 2). All qubit properties in this initial tune-up including the drive gate of each qubit used for randomized benchmarking are indicated in Supplementary Note 3. An in-depth noise analysis detailed in ref. ^21^ indicates that the qubit performance is bounded by a hyperfine-limited ${T}_{{{{\rm{2}}}}}^{*}$ of approximately 2 μs arising from the out-of-plane component of the magnetic field, which could be alleviated by using purified germanium^26,27^. We also demonstrate tunable exchange interactions between neighboring qubit pairs (see Supplementary Note 4), making this spin qubit array representative for future quantum processors based on densely occupied QD arrays. Supplementary Note 15 discusses how error correction codes could be performed on such a device.

### Qubit drive efficiency and tunability

**Fig. 2:**
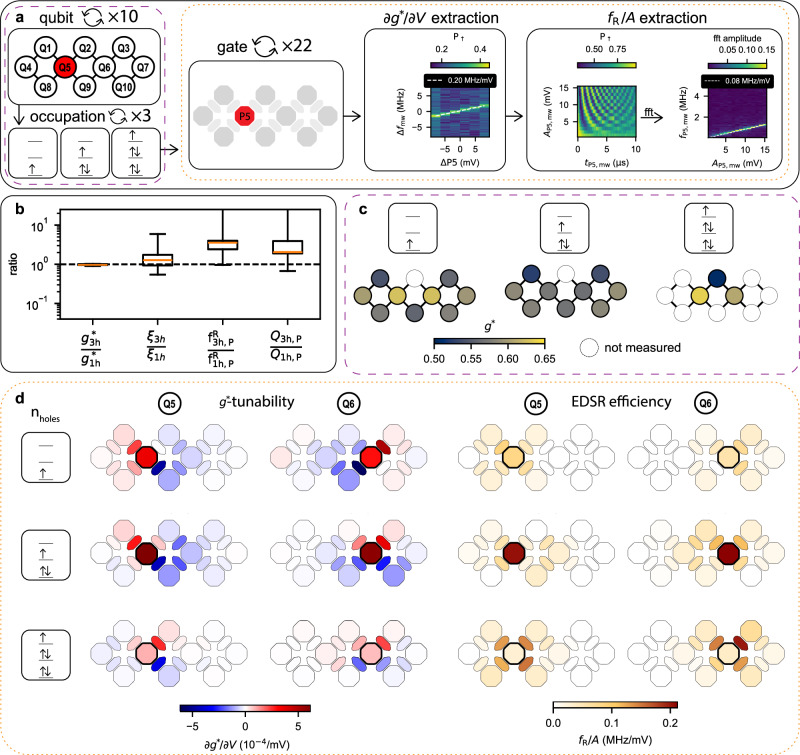
Statistical analysis of the 10-spin qubit array. **a** Flow diagram of g^*^-tunability and driving efficiency extraction. After selecting one of the qubits, Q1–Q10, and looping over the one-, three-, and five-hole occupation, the g^*^-tunability and drive efficiencies are extracted sequentially for each of the 10 plunger and 12 barrier gates. For the g^*^-tunability each of the gates is scanned in the range of  ±8 mV while sweeping the microwave frequency across the corresponding qubit frequency on a dedicated fixed gate. By fitting the peak in the recorded signal, the frequency slope can be determined in MHz/mV, which can be converted into a g-factor slope in 1/mV. For the driving efficiency the amplitude is swept from 1 to 15 mV, while applying a microwave pulse on each of the gates. By performing a fast-Fourier transform and fitting the dominant frequency contribution with a linear fit, the driving slope in MHz/mV can be extracted. **b** Boxplots in Spear style containing the ratios of g^*^, LSES, driving efficiency, and quality factor in the three- and one-hole occupation for plunger drive. Each boxplot contains 9 data points with all 10 qubits except Q2. The orange line denotes the median value and the whiskers are from the first quartile to third quartile. **c** g^*^ of all 10 qubits in the one-, three-, and five-hole occupation. **d** g^*^-tunability and driving efficiency for qubit Q5 and Q6 as a function of all 10 plunger and 12 barrier gates. Each row corresponds to a different hole occupation of one, three, and five respectively.

This two-dimensional 10-qubit array provides a sufficiently large and robust platform to gather a comprehensive dataset on the effects of varying qubit sites and hole occupancies while avoiding device-to-device variability. By systematically performing the measurement protocol shown in Fig. 2a, we characterize the LSES and driving efficiency *f*_R_/*A*, with *f*_R_ the Rabi frequency and *A* the applied drive amplitude on the device level, across all qubits with one-, three-, and five-hole occupancy.

These driving properties are intimately linked to the sensitivity of the g-tensor to the electrostatics and its environment^28,29^. Indeed, Rabi oscillations are governed by modulation of the transverse component of the g-tensor through AC gate voltages, while the LSES measures the gate ability to tune the longitudinal component, which influences qubit coherence in the charge-noise-limited regime via ${T}_{2}^{*}\propto 1/\xi$ with $\xi=\sqrt{{\sum }_{{{{\rm{gate}}}}}{(\partial {g}^{*}/\partial {V}_{{{{\rm{gate}}}}})}^{2}}$^12,30^. Here, the effective g-factor for a given magnetic field is expressed as ${g}^{*}=| {{{\bf{g}}}}\overrightarrow{b}|$, with **g** representing the g-tensor and $\overrightarrow{b}=\overrightarrow{B}/| \overrightarrow{B}|$ the normalized magnetic field direction. The total longitudinal susceptibility, *ξ*, encompasses all the gates of the device and assumes uncorrelated noise contributions between them. The interplay between driving efficiency and longitudinal susceptibility can be captured by a qubit quality factor, defined as $Q={f}_{{{{\rm{R}}}}}/\xi \propto {f}_{{{{\rm{R}}}}}\cdot {T}_{2}^{*}$^31^. The quality factor *Q* enables the identification of operational sweet spots and their dependence on hole configurations.

Figure 2b summarizes qubit statistics collected across the 10 qubits for single- and three-hole occupations, visualizing their ratios in a boxplot. We refer to plunger drive when the qubit is driven with a plunger gate, and barrier drive when a barrier gate is used. For the drive efficiency, we only focus on the plunger drive for each qubit. As the hole occupancy increases from one to three, both g^*^ and *ξ* show minimal variation, while the plunger drive efficiency improves by a median factor of 3.6. With a modest median increase of 1.3 in *ξ*, the quality factor improves by a median factor of 2.0. Here, we refer to the median values, as the average is skewed by Q3, which is barely driven by the plunger gate in the single-hole occupation regime. Notably, the whisker representing the Rabi frequency and quality factor ratio for single- and three-hole occupancies extends towards infinity, as no measurable driving of Q3 using the plunger gate P3 was observed in the single-hole occupancy within the applied voltage amplitude range, which confirms the importance of investigating the gate and hole occupation dependence of EDSR in large qubit arrays.

The underlying data of the g-factor variability is visualized in Fig. 2c across different hole occupancies and qubit sites. Despite holes in germanium having a large g-tensor anisotropy, we find a small relative variation in g-factors across 10 qubits within a single device and different hole configurations, with an average g-factor of 0.58 ± 0.03. We believe that this small variability results from the large contribution of the perpendicular component of the g-tensor when the magnetic field is oriented out-of-plane. Additional considerations in terms of variability can be found in Supplementary Note 14.

Exemplary data for the central qubits, Q5 and Q6, which are measured across one-, three- and five-hole occupancies, are shown in Fig. 2d. The data show a distinct increase in qubit plunger drive as the hole occupation increases from one to three, while the efficiency of the barriers remains approximately unchanged. However, increasing the hole occupation to five reverses this pattern, with the qubit plunger drive becoming significantly weaker while the barrier drives become stronger. The observed g^*^-tunability patterns include barriers exhibiting both negative and positive ∂g^*^/∂*V*_gate_, but the relative positions of these barriers do not reveal clear trends across the full array, making it challenging to identify their origin. Generally, barriers along the diagonal often have approximately opposite values, e.g., for Q5 barrier gates B2 and B9 have similar magnitude but opposite sign for the single and three-hole occupation, while B3 and B8 are both weak. The ∂g^*^/∂*V*_gate_ value associated with the qubit plunger gate is always positive, but its magnitude is comparable to that of the associated barriers. These patterns are supported and further explained by numerical simulations in Supplementary Note 6. The trend of increased top plunger driving efficiency from one to three holes is observed in eight of the nine measured qubits (Q2 has only been measured in the five-hole regime). The exception is qubit Q4, which exhibits a constant plunger drive efficiency from one- to three-hole occupation. The complete dataset for g^*^-tunability and driving efficiency across all qubits and gates is provided in Supplementary Notes 7 and 8. Overall, the three-hole regime is a more favorable regime for operation, as the driving mechanisms are more robust, with importantly no instances of zero driving, unlike in the single-hole regime.

### Modeling of single- and multi-hole quantum dots

The improvement of driving efficiency in the three-hole regime is captured by simulations of a realistic geometry using a four-band Luttinger-Kohn Hamiltonian and full configuration interaction for the Coulomb correlations. These simulations help understanding the trends of the LSES and Rabi frequency shown in Fig. 2 (see also Supplementary Note 6).

Our analytical and numerical modeling suggests that the enhancement of the driving efficiency of the plunger gate in the three-hole regime results from the interplay between symmetry breakings and Coulomb interactions. In the single-hole regime, the ground state is a spin doublet with a quasi-circular *s*-like envelope. When driven by the plunger gate, the radius of the envelope oscillates, but its shape remains circular resulting in an isotropic response of the in-plane g-factors. This modulates the longitudinal (LSES) component much stronger than the transverse (Rabi) component of the Larmor vector ${{{\bf{g}}}}\cdot \overrightarrow{b}$. This could be the reason why Q3 could not be driven by the plunger in the single-hole occupation.

In the non-interacting three-hole regime, the ground state doublet is an elongated *p*-like orbital *p*_**u**_ whose in-plane axis **u** is oriented along the direction of weakest confinement. It is energetically split from an orthogonal *p*_**v**_ orbital by bias and disorder-induced asymmetries, giving rise to a well-defined qubit. This *p*_**u**_ orbital as well as its response to the plunger gate is much more anisotropic, enabling stronger transverse coupling and thus faster Rabi oscillations. The same argument could also explain a decrease in Rabi frequency of the plunger gate in the five-hole regime. The next spin doublet now occupies the *p*_**v**_ orbital, which is, however, less responsive to the plunger gate because its axis is oriented along a stronger confinement direction. The shape of the spin wave-function is less relevant for the barrier gates, whose highly anisotropic potentials can couple states with any symmetry.

The above considerations, drawn in the non-interacting limit, can be extended to the interacting case. The Rabi frequency can generally be split into single-particle (SP) and many-body (MB) contributions: ${f}_{{{{\rm{R}}}}}=\big| \, {\vec{f}}_{R}^{SP}+{\vec{f}}_{R}^{MB} \big|$. The latter depends on the strength of the Coulomb interactions and the gap between the single-particle orbitals. By mixing orbital configurations together, the Coulomb interactions reshape the hole density and the response of the ground state doublet to the drive fields. Interestingly, the MB contributions can be mapped to a disordered Hubbard spin model, where the sites correspond to the orbital states. Consequently, the Rabi driving could be enhanced, similar to flopping-mode qubits^32^ (see Supplementary Note 6C). In general, we find that our full configuration interaction simulations still match the trends of the non-interacting model but reveal that the Coulomb interactions have a significant quantitative impact on the Rabi frequencies, especially for barrier gates.

**Fig. 3:**
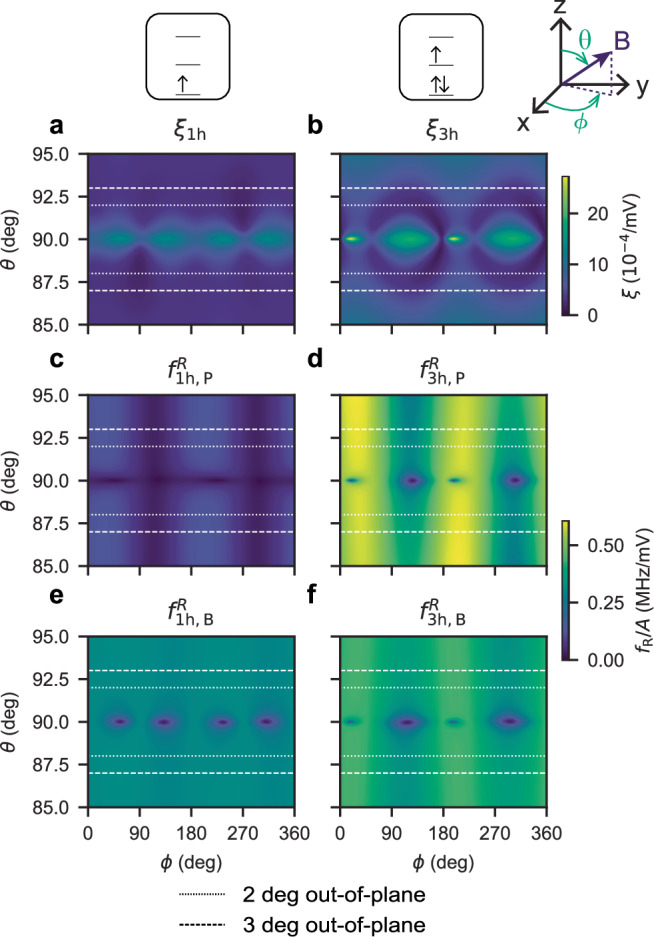
Simulated *ξ* and Rabi frequency *f*_R_ as a function of magnetic field angle at 41 mT in single- and three-hole occupancies. Data have been obtained through full configuration interaction simulations. **a** *ξ* in the single-hole occupation, **b** *ξ* in the triple-hole occupation, **c** *f*_R_ in the single-hole occupation when driving with the top plunger, **d** *f*_R_ in the triple-hole occupation when driving with the top plunger, **e** *f*_R_ in the single-hole occupation when driving with the most efficient barrier, **f** *f*_R_ in the triple-hole occupation when driving with the most efficient barrier. The dashed and dotted lines indicate the estimated operation field angle of the experiments.

Maps of simulated *ξ* and Rabi frequencies for the plunger gate and one of the barrier gates are shown in Fig. 3 as a function of the magnetic field orientation. They highlight the increase of the efficiency of the plunger gate in the three-hole regime, and the strong dependence of all quantities on the azimuthal angle *ϕ* when the magnetic field goes in-plane (*θ* = 90°). In particular, the driving efficiency approaches zero while *ξ* is large (hence ${T}_{2}^{*}$ small) at specific angles *ϕ*. The device-to-device variability of these angles resulting from bias conditions is a considerable challenge for the tuning and calibration of large-scale systems. We envision that imprinting an initial anisotropy, strain or potential can help in reducing these variations.

The simulations thus clearly show the benefits of avoiding the region around *θ* = 90° and setting the magnetic field slightly out-of-plane in the region indicated by the dashed lines. The Rabi frequency of the plunger gate is sufficiently strong and more uniform in *ϕ* in the three-hole regime, while the charge susceptibility is much improved (smaller *ξ*). Consequently, driving three-hole qubits with the plunger gate and magnetic field slightly out-of-plane may provide a reliable and uniform operation mode for scaling up to large arrays in nuclear spin-free purified germanium^26,27^.

### Driving locality in extended qubit arrays

EDSR is commonly expected to be a local driving mechanism due to the localized nature of the electric field^8,33,34^. However, the locality of EDSR may be altered by electric crosstalk, which can arise from capacitive coupling between gates or electric field propagating in the heterostructure. Here, we aim to assess the extent of this locality. To do so, we analyze the acquired data by focusing on the driving efficiency of each gate to each qubit. Specifically, we evaluate the driving locality when applying a microwave pulse to any of the 22 available plunger and barrier gates. In this analysis, we consider four distinct cases involving either barrier or plunger drive and either one- or three-hole occupation. The five-hole occupation is excluded in this study due to insufficient data across the array. For each driving gate, the corresponding target qubit is defined as the qubit closest to the driving gate. We note that the plunger gates are patterned after the barrier gates and they are separated by a 10 nm thick oxide layer (see Method section).

**Fig. 4:**
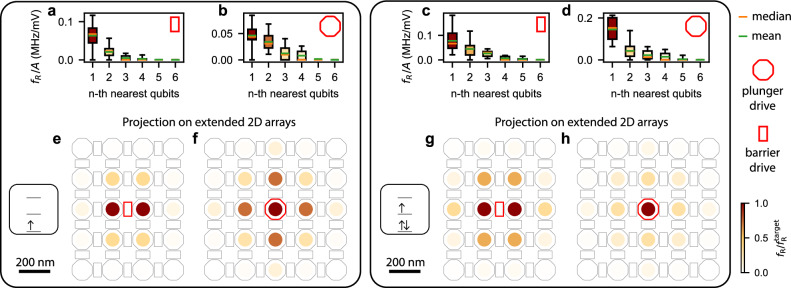
Rabi driving locality. **a**–**d** Boxplots in Spear style depicting the Rabi driving efficiency for all gates up to the sixth nearest qubit with indicated mean (green line) and median value (orange line). Data are shown for **a** barrier drive in the single-hole occupation, **b** plunger drive in the single-hole occupation, **c** barrier drive in the three-hole occupation, and **d** plunger drive in the three-hole occupation. The color of each boxplot represents the Rabi frequency normalized to that of the target qubit. **e**–**h** Projection of the normalized Rabi frequency onto an extended, densely populated two-dimensional spin qubit array. Data are shown for barrier drive in the single-hole occupation (**e**), plunger drive in the single-hole occupation (**f**), barrier drive in the three-hole occupation (**g**), and plunger drive in the three-hole occupation (**h**).

In Fig. 4, driving efficiency is categorized by the physical distance of each gate to each qubit. Independent of direction, we then define nearest neighbors based on this physical proximity. Driving efficiency is quantified by the averaged results for all *n*-th nearest qubits over all driving gates. Figure 4a–d presents the Rabi driving efficiencies as boxplots up to the sixth nearest neighbor for both barrier and plunger drive, with a corresponding maximum physical distance of 550 nm in the device plane (all distances of the *n*-th nearest qubits and their ranks are listed in Supplementary Note 11). To evaluate drive locality, we express our data in terms of the normalized driving efficiency ${f}_{{{{\rm{R}}}}}/{f}_{{{{\rm{R}}}}}^{{{{\rm{target}}}}}$, obtained by normalizing the Rabi driving efficiency relative to that of the target qubit. Lower normalized driving efficiencies for distant qubits indicate less cross-talk and more localized driving. For qubits located beyond the sixth nearest neighbor, driving efficiency falls below 0.01 MHz/mV, which is below the sensitivity of the measurement within the range of applied drive amplitudes. We generally observe a decrease in driving efficiency for larger distances in both one- and three-hole cases, for both plunger and barrier drives. The drop in mean driving efficiency from the first to the second nearest qubit is largest for the three-hole plunger drive and single-hole barrier drive. These results are compatible with the expected electric field decay as further discussed in Supplementary Note 12.

To illustrate the impact of cross-talk, we project the measured results onto an extended densely populated two-dimensional spin qubit array. When driving a target qubit with a specific gate, the *n*-th nearest qubits are color-coded according to the mean of the normalized Rabi efficiency measured experimentally and presented in the boxplots of Fig. 4a–d. This visualization highlights how much each qubit would be affected when driven using a plunger or barrier gate under single- and three-hole occupations.

Figure 4e, g depicts the projected cross-talk for barrier driving. By design, barrier gates drive the two nearest qubits equally, leading to pronounced cross-talk between them. Beyond the first nearest qubits, the single-hole occupation exhibits significantly reduced cross-talk. Figure 4f, h illustrates the projected cross-talk for plunger driving. In the three-hole regime, cross-talk is small. In contrast, in the single-hole regime, plunger driving can result in negligible or even vanishing driving efficiencies, requiring large drive amplitudes and consequently inducing substantial cross-talk.

Comparing the different regimes, the most localized driving is observed for plunger driving in the three-hole regime and barrier driving in the single-hole regime. Among these, the former achieves minimal cross-talk while maintaining the highest driving efficiencies, making it here the most favorable driving scheme in terms of efficiency and cross-talk mitigation in a two-dimensional array with dense occupation. In Supplementary Note 13, we further estimate that this operational regime enables the control of extended 2D arrays by engineering repeating tiles that require only a limited number of unique Larmor frequencies across the entire array.

## Discussion

Our results demonstrate that despite the strong sensitivity of the highly anisotropic germanium g-tensor, remarkably uniform qubit characteristics can be obtained in a 10-qubit array. We have observed an enhanced and highly localized drive when manipulating qubits in the three-hole occupation regime using the plunger gate, consistent across the entire array. Numerical and analytical simulations reinforce these findings, highlighting the impact of *p*-like orbital anisotropy and intra-dot Coulomb interactions in the multi-hole regime. For future quantum processors based on spin qubits in strained Ge/SiGe quantum wells, we envision that an effective approach may be to orient the magnetic field slightly out of plane to avoid weak spots and ensure robust, localized control, as demonstrated here. Our work also highlights the importance of including parameters such as the hole occupation and drive gate into the design considerations of large qubit arrays.

## Methods

### Fabrication

The device is fabricated on a Ge/SiGe heterostructure with a 16 nm germanium quantum well buried 55 nm below the semiconductor/oxide interface on a germanium substrate, as described in ref. ^21^. The ohmic contacts are created first through platinum deposition and diffusion. For the gate stack, the barrier gate layer (20 nm thick) is deposited, followed by the screening gate layer (30 nm thick), and finally the plunger layer (40 nm thick). All the gate layers are made of palladium, deposited at room temperature. The barrier gates are separated from the heterostructure by a 7 nm thick aluminum oxide and a 5 nm oxide is deposited after the barrier and the screening layers.

### Experimental setup

The sample was mounted on a printed circuit board (PCB) with 100 direct current (DC) lines. Thirty lines include a resistive bias network combining alternating current (AC) and DC signals. The AC input is capacitively coupled (100 nF) and referenced to ground through 100 kΩ, with a 1 MΩ resistor linking AC and DC nodes. The DC bias is decoupled to ground via 100 pF. All plunger, barrier, and sensor plunger gates are bonded to AC lines.

Experiments were performed in a Bluefors LD400 dilution refrigerator with a base temperature of a few tens of millikelvin. A superconducting solenoid magnet operated in driven mode provided an axial magnetic field. The PCB was mounted on a custom cold finger at a position slightly off-centered, which reduced the magnetic field amplitude at the sample by a factor of 0.69 relative to the nominal field recorded in the metadata.

DC lines were filtered at multiple stages of the refrigerator and connected outside the cryostat to a battery-powered SPI rack for voltage control. AC lines were attenuated within (9–15 dB) and outside (~10 dB) the dilution fridge, and connected to a Keysight M3202A arbitrary waveform generator cluster. Instrument control was implemented using pulselib (pulse-lib.readthedocs.io) and internal qconstruct libraries. Virtual gates have been used to account for capacitive gate-to-gate crosstalk (see Supplementary Note 5).

The device incorporated four single-hole transistors (SHTs) at its edges, each consisting of a source ohmic, source barrier, sensor plunger, drain barrier, and drain ohmic. The barriers controlled tunneling between the reservoir and the sensor quantum dot. Coulomb peaks appeared in transport before the SHT current fully turned on; biasing on the flank of a peak enabled single-charge detection.

Charge sensing employed reflectometry. The PCB feedline was coupled to four LC tank circuits (100 pF capacitors with off-chip inductors of a few μH). A 5 kΩ shunt resistor set an RC constant *τ* = 0.5 μs. The input RF tone was attenuated by 43 dB in the fridge and by a similar amount at room temperature. Reflected signals were amplified 35 dB by a cryogenic low-noise amplifier at 4 K, digitized at room temperature with a Keysight M3102A module, and demodulated using its FPGA.

Each SHT source was bonded to one tank circuit, with the drain grounded. Resonance frequencies ranged from 70–170 MHz. Resistance changes induced by nearby charges modified the reflected amplitude; the RF phase was chosen to maximize sensitivity.

Single-shot readout was performed by repeating pulse sequences *n* times and assigning binary outcomes depending on whether the measured amplitude crossed a calibrated threshold. Thresholds were set from histograms of the two charge states, with insufficient separation contributing to readout errors. Typically, 500 repetitions were used per measurement.

### Read-out

We perform parity read-out of qubit pairs via Pauli spin blockade. To do so, we pulse from the (1,1) charge symmetry point to the PSB region at the (1,1)-(2,0) anti-crossing, such that we map odd parity to (2,0) and even parity to (1,1). The charge signal is measured via RF-reflectometry. Before measuring, we let the system settle 0.5 μs to avoid drift of the charge signal. Since the system has been re-tuned for various charge configurations, visibilities and integration times do vary. Exemplary values for each qubit during randomized benchmarking can be found in Supplementary Note 1.

## Supplementary information

Supplementary Information

Transparent Peer Review file

## Data Availability

The data supporting the findings of this study are openly available in the 4TU.ResearchData repository under the 10.4121/5ee5b0d3-e838-478e-990d-02c50b75eeab.

## References

[1] Hendrickx, N. W. et al. A four-qubit germanium quantum processor. *Nature* **591**, 580–585 (2021). 33762771 10.1038/s41586-021-03332-6

[2] Philips, S. G. J. et al. Universal control of a six-qubit quantum processor in silicon. *Nature* **609**, 919–924 (2022). 36171383 10.1038/s41586-022-05117-x PMC9519456

[3] Zhang, X. et al. Universal control of four singlet–triplet qubits. *Nat. Nanotechnol.* https://www.nature.com/articles/s41565-024-01817-9 (2024). 10.1038/s41565-024-01817-9 PMC11835736 39482413

[4] Thorvaldson, I. et al. Grover’s algorithm in a four-qubit silicon processor above the fault-tolerant threshold. *Nat. Nanotechnol.* **20**, 472–477 (2025). 39979400 10.1038/s41565-024-01853-5 PMC12014505

[5] Borsoi, F. et al. Shared control of a 16 semiconductor quantum dot crossbar array. *Nat. Nanotechnol.* **19**, 21–27 (2024). 37640909 10.1038/s41565-023-01491-3 PMC10796274

[6] Wang, C.-A. et al. Operating semiconductor quantum processors with hopping spins. *Science* **385**, 447–452 (2024). 39052794 10.1126/science.ado5915

[7] George, H. C. et al. 12-spin-qubit arrays fabricated on a 300 mm semiconductor manufacturing line. *Nano Lett.* **25**, 793–799 (2025). 39721970 10.1021/acs.nanolett.4c05205 PMC11741134

[8] Bulaev, D. V. & Loss, D. Spin relaxation and decoherence of holes in quantum dots. *Phys. Rev. Lett.* **95**, 076805 (2005). 16196813 10.1103/PhysRevLett.95.076805

[9] Bulaev, D. V. & Loss, D. Electric dipole spin resonance for heavy holes in quantum dots. *Phys. Rev. Lett.* **98**, 097202 (2007). 17359191 10.1103/PhysRevLett.98.097202

[10] Scappucci, G. et al. The germanium quantum information route. *Nat. Rev. Mater.* **6**, 926–943 (2020).

[11] Wang, Z. et al. Optimal operation points for ultrafast, highly coherent Ge hole spin-orbit qubits. *npj Quantum Inf.* **7**, 54 (2021).

[12] Hendrickx, N. W. et al. Sweet-spot operation of a germanium hole spin qubit with highly anisotropic noise sensitivity. *Nat. Mater.* **23**, 920–927 (2024). 38760518 10.1038/s41563-024-01857-5 PMC11230914

[13] Carballido, M. J. et al. Compromise-free scaling of qubit speed and coherence. *Nat. Commun.* **16**, 7616 (2025). 40817078 10.1038/s41467-025-62614-z PMC12356937

[14] Bassi, M. et al. Optimal operation of hole spin qubits. *arXiv* http://arxiv.org/abs/2412.13069 (2024).

[15] Abadillo-Uriel, J. C., Rodríguez-Mena, E. A., Martinez, B. & Niquet, Y.-M. Hole-spin driving by strain-induced spin-orbit interactions. *Phys. Rev. Lett.* **131**, 097002 (2023). 37721821 10.1103/PhysRevLett.131.097002

[16] Mauro, L., Rodríguez-Mena, E. A., Bassi, M., Schmitt, V. & Niquet, Y.-M. Geometry of the dephasing sweet spots of spin-orbit qubits. *Phys. Rev. B* **109**, 155406 (2024).

[17] Liles, S. D. et al. Spin and orbital structure of the first six holes in a silicon metal-oxide-semiconductor quantum dot. *Nat. Commun.* **9**, 3255 (2018). 30108212 10.1038/s41467-018-05700-9 PMC6092405

[18] Jin, I. K. et al. Probing g-tensor reproducibility and spin-orbit effects in planar silicon hole quantum dots. *arXiv* http://arxiv.org/abs/2411.06016 (2024).

[19] Leon, R. C. C. et al. Coherent spin control of s-, p-, d- and f-electrons in a silicon quantum dot. *Nat. Commun.* **11**, 797 (2020). 32047151 10.1038/s41467-019-14053-w PMC7012832

[20] Mai, P. Y. et al. Enhancement of electric drive in silicon quantum dots with electric quadrupole spin resonance. *arXiv* http://arxiv.org/abs/2502.01040 (2025).

[21] Stehouwer, L. E. A. et al. Exploiting strained epitaxial germanium for scaling low-noise spin qubits at the micrometre scale. *Nat. Mater.* https://www.nature.com/articles/s41563-025-02276-w (2025). 10.1038/s41563-025-02276-w PMC12657214 40619566

[22] Stehouwer, L. E. A. et al. Germanium wafers for strained quantum wells with low disorder. *Appl. Phys. Lett.* **123**, 92101 (2023).

[23] Ono, K., Austing, D. G., Tokura, Y. & Tarucha, S. Current rectification by Pauli exclusion in a weakly coupled double quantum dot system. *Science* **297**, 1313–1317 (2002). 12142438 10.1126/science.1070958

[24] Fransson, J. & Råsander, M. Pauli spin blockade in weakly coupled double quantum dots. *Phys. Rev. B* **73**, 205333 (2006).

[25] Knill, E. et al. Randomized benchmarking of quantum gates. *Phys. Rev. A* **77**, 012307 (2008).

[26] Sigillito, A. et al. Electron spin coherence of shallow donors in natural and isotopically enriched germanium. *Phys. Rev. Lett.* **115**, 247601 (2015). 26705654 10.1103/PhysRevLett.115.247601

[27] Moutanabbir, O. et al. Nuclear spin-depleted, isotopically enriched 70Ge/28Si70Ge quantum wells. *Adv. Mater.* **36**, 2305703 (2024). 10.1002/adma.202305703 38009242

[28] Crippa, A. et al. Electrical spin driving by g-matrix modulation in spin-orbit qubits. *Phys. Rev. Lett.* **120**10.1103/PhysRevLett.120.137702 (2017). 10.1103/PhysRevLett.120.137702 29694195

[29] Michal, V. P., Venitucci, B. & Niquet, Y.-M. Longitudinal and transverse electric field manipulation of hole spin-orbit qubits in one-dimensional channels. *Phys. Rev. B* **103**, 045305 (2021).

[30] Piot, N. et al. A single hole spin with enhanced coherence in natural silicon. *Nat. Nanotechnol.* **17**, 1072–1077 (2022). 36138200 10.1038/s41565-022-01196-z PMC9576591

[31] Stano, P. & Loss, D. Review of performance metrics of spin qubits in gated semiconducting nanostructures. *Nat. Rev. Phys.* **4**, 672–688 (2022).

[32] Benito, M. et al. Electric-field control and noise protection of the flopping-mode spin qubit. *Phys. Rev. B* **100**, 125430 (2019).

[33] Golovach, V. N., Borhani, M. & Loss, D. Electric-dipole-induced spin resonance in quantum dots. *Phys. Rev. B* **74**, 165319 (2006).

[34] Nowack, K. C., Koppens, F. H. L., Nazarov, Y. V. & Vandersypen, L. M. K. Coherent control of a single electron spin with electric fields. *Science* **318**, 1430–1433 (2007). 17975030 10.1126/science.1148092

