# Supplementary material for: Robust and localised control of a 10-spin qubit array in germanium
Source: Nat Commun. 2025 Nov 26;16:10560. doi: 10.1038/s41467-025-65577-3 (PMC12657959; doi:10.1038/s41467-025-65577-3)
Supplement: Supplementary file 1 — Supplementary Information [file 41467_2025_65577_MOESM1_ESM.pdf]

CONTENTS

Suppl. Note 1. Extended methods S2
A. raw SEM image S2
B. Read-out visibilities S2

Suppl. Note 2. Single-qubit gate randomised benchmark S3

Suppl. Note 3. Qubit properties in the initial hole configuration S5

Suppl. Note 4. Exchange interaction in the 10-qubit array S5

Suppl. Note 5. Gate virtualisation S5

Suppl. Note 6. Modeling of single- and multi-hole quantum dots S6
A. Models and device S6
B. Single-hole dots S7
C. Three and five-hole dots S10
1. Analytical expressions S11
2. Numerical simulations S14

Suppl. Note 7. EDSR driving efficiency S17

Suppl. Note 8. g-factor tunability S18

Suppl. Note 9. LSES extraction S19

Suppl. Note 10. Driving efficiency extraction S22

Suppl. Note 11. Physical distance from gates to qubits S26

Suppl. Note 12. Spatial locality of the electric field and qubit drive S27

Suppl. Note 13. Frequency cross-talk S29

Suppl. Note 14. Scalability considerations and variability S30

Suppl. Note 15. Error correction S30

References S31

Suppl. Note 1. EXTENDED METHODS

A. raw SEM image

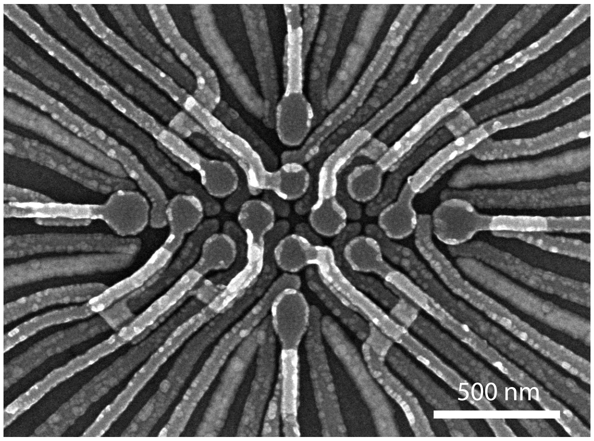

Supplementary Figure S1. Scanning electron microscope image of a device nominally identical to the one utilised in the experiments without any false colouring.

B. Read-out visibilities

Since the system has been re-tuned for various charge configurations, visibilities and integration times do vary. Exemplary values for each qubit during randomised benchmarking are given in the following table:

Supplementary Table S1. Read-out visibilities and integration times.

	**Q1**	**Q2**	**Q3**	**Q4**	**Q5**	**Q6**	**Q7**	**Q8**	**Q9**	**Q10**
**PSB pair**	Q1,Q4	Q2,Q6	Q3,Q7	Q1,Q4	Q5,Q9	Q2,Q6	Q3,Q7	Q4,Q8	Q5,Q9	Q7,Q10
**Sensor**	S_{West}	S_{North}	S_{East}	S_{West}	S_{South}	S_{North}	S_{East}	S_{West}	S_{South}	S_{East}
**Visibility**	0.44	0.66	0.68	0.49	0.66	0.62	0.85	0.68	0.58	0.50
t_{measure}(µs)	12	6	5	15	8	6	5	8	12	3
t_{settle}(µs)	0.5	0.5	0.5	0.5	3	0.5	0.5	0.5	0.5	0.5

While the readout visibility is currently limited, this is due to the lack of readout parameter optimization, which can decrease SPAM errors significantly. Improved visibility could be achieved by refining ramp times, and initialisation sequences such as larger exchange coupling during initialisation.

Suppl. Note 2. SINGLE-QUBIT GATE RANDOMISED BENCHMARK

We perform randomised benchmarking (RB) to measure the single-qubit gate fidelity of the 10 qubits in the initial hole configuration, outlined in Table **S5**, using the set of Clifford gates defined in Table **S2**. Details about this Clifford group can be found in Ref. [S1].

Supplementary Table S2. Single-qubit Clifford sequence and their composition. $X_{\pi/2}$ and $Z_{\pi/2}$ are referring to $\pi/2$ rotation around the x-axis and the z-axis, respectively, of the Bloch sphere of a single-qubit. The average number of elementary gates per Clifford composition is 2 as the Z rotation is generated by a change of qubit's reference frame in software, which makes it error-free.

Clifford	Composition
C_1	$X_{\pi/2}X_{-\pi/2}$
C_2	$X_{\pi/2}X_{\pi/2}$
C_3	$Z_{-\pi/2}X_{\pi/2}X_{\pi/2}Z_{\pi/2}$
C_4	$X_{\pi/2}Z_{\pi/2}Z_{\pi/2}X_{\pi/2}$
C_5	$X_{\pi/2}Z_{-\pi/2}X_{\pi/2}Z_{\pi/2}$
C_6	$X_{\pi/2}Z_{\pi/2}X_{\pi/2}Z_{\pi/2}$
C_7	$X_{-\pi/2}Z_{-\pi/2}X_{\pi/2}Z_{\pi/2}$
C_8	$X_{-\pi/2}Z_{\pi/2}X_{\pi/2}Z_{-\pi/2}$
C_9	$Z_{-\pi/2}X_{\pi/2}Z_{\pi/2}X_{\pi/2}$
C_{10}	$Z_{-\pi/2}X_{\pi/2}Z_{\pi/2}X_{-\pi/2}$
C_{11}	$Z_{\pi/2}X_{\pi/2}Z_{-\pi/2}X_{\pi/2}$
C_{12}	$Z_{\pi/2}X_{\pi/2}Z_{-\pi/2}X_{-\pi/2}$
C_{13}	$Z_{-\pi/2}X_{\pi/2}Z_{\pi/2}X_{\pi/2}Z_{-\pi/2}$
C_{14}	$Z_{\pi/2}X_{-\pi/2}Z_{-\pi/2}X_{-\pi/2}Z_{\pi/2}$
C_{15}	$X_{\pi/2}Z_{\pi/2}X_{-\pi/2}$
C_{16}	$X_{\pi/2}Z_{-\pi/2}X_{-\pi/2}$
C_{17}	$X_{-\pi/2}Z_{\pi/2}Z_{\pi/2}X_{-\pi/2}Z_{-\pi/2}$
C_{18}	$X_{-\pi/2}Z_{-\pi/2}Z_{-\pi/2}X_{-\pi/2}Z_{\pi/2}$
C_{19}	$X_{\pi/2}Z_{-\pi/2}X_{\pi/2}$
C_{20}	$X_{\pi/2}Z_{\pi/2}X_{\pi/2}$
C_{21}	$Z_{-\pi/2}X_{\pi/2}Z_{\pi/2}X_{\pi/2}Z_{\pi/2}$
C_{22}	$Z_{-\pi/2}X_{\pi/2}Z_{\pi/2}X_{-\pi/2}Z_{-\pi/2}$
C_{23}	$X_{\pi/2}X_{\pi/2}Z_{\pi/2}$
C_{24}	$X_{-\pi/2}X_{-\pi/2}Z_{-\pi/2}$

The randomised benchmarking data for all 10 qubits are shown in Figure **S2**. We assume an exponential decay of the form $P_{\text{even}} = aF^n + b$ where F is the circuit level fidelity, n is the number of Clifford operations, a and b are fitting parameters depending on the state preparation and measurement. The Clifford fidelity is then given by

$$F_{\text{C}} = 1 - (1 - F)/2, \tag{1}$$

and the native gate fidelity is

$$F_{\text{gate}} = 1 - (1 - F_{\text{C}})/(2N_{\text{avg}}), \tag{2}$$

where N_{avg} is the average gate number per Clifford, which for our chosen Clifford set is 2 [S1]. For each of these datasets we have probed up to either 100, 200, or 400 numbers of Clifford gates in either 100 or 199 steps. We use for each Clifford gate number 10 randomisations. The gate fidelity and its corresponding standard deviation are calculated using maximum likelihood estimation (MLE). The standard deviation here captures the statistical error of the fit following MLE, and does not take into account systematic errors. To capture these coming e.g. from drifts over time or gate-dependent depolarising noise one would need to perform more elaborated measurements such as blind RB or gate set tomography [S2, S3]. We estimate the systematic error to be of the order of 0.1%, i.e. the systematic error is likely dominant over the small statistical error. Hence, the gate fidelities given in Figure 1c of the main manuscript are represented in discrete steps of 0.1%, while the fits in Figure **S2** are shown with the statistical error.

Table **S3** and Table **S4** show the expected (obtained from Rabi oscillations just before the RB measurement) and measured offset and amplitude for the RB decay. The fits are close to the expected fitting parameters. Particularly, the fits for Q1, Q2, and Q8, which all do not reach saturation have very good agreement between the expected and measured amplitude and offset. We also plot the decay curve for a gate fidelity of 99% and 99.9% using the same

amplitude and offset as obtained from the maximum likelihood estimation. The data shows that all single qubit gate fidelities of the ten qubits are clearly above 99%.

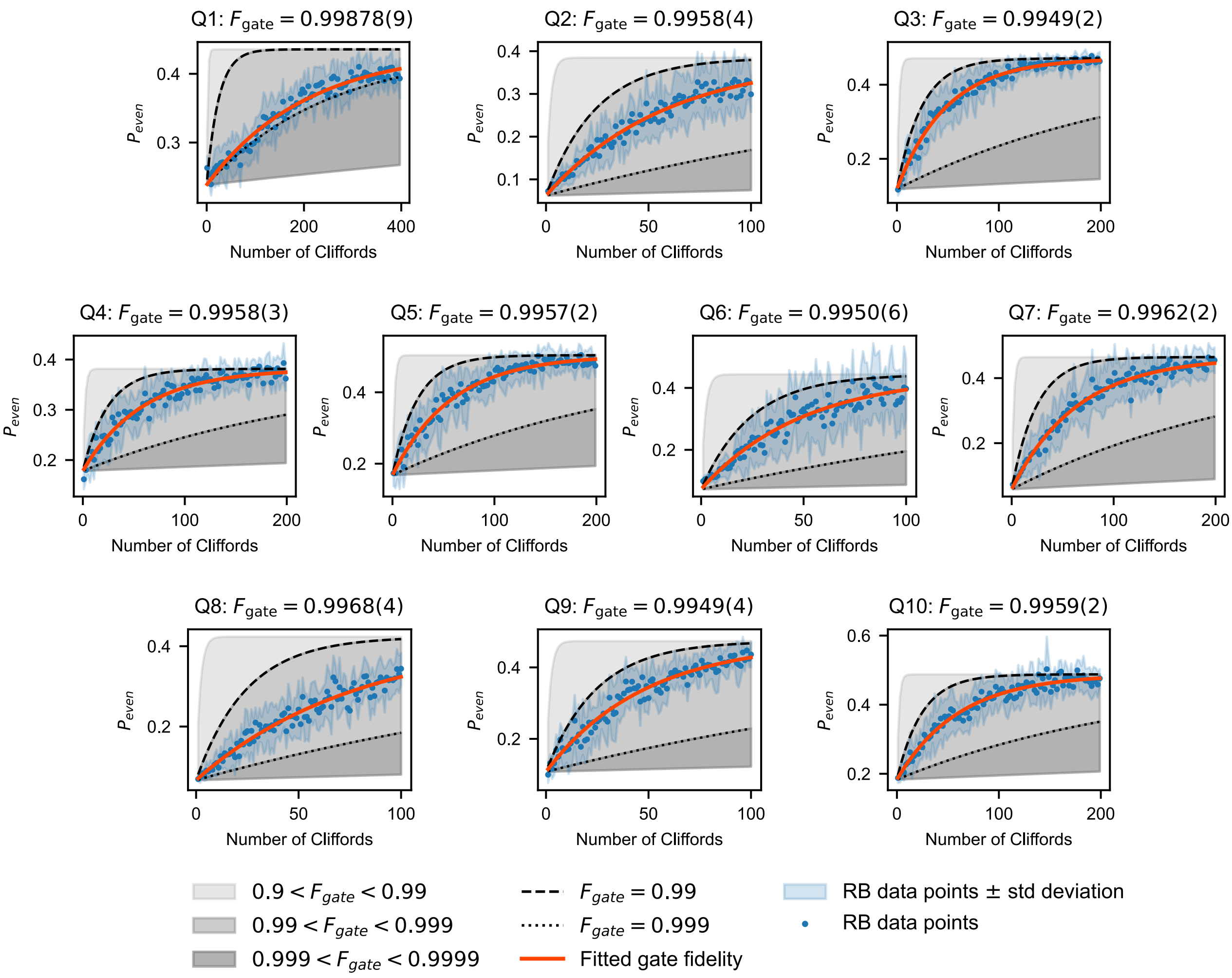

Supplementary Figure S2. Single-qubit gate benchmarking on the 10 qubits. The black dots correspond to the averaged randomised benchmarking data over 10 randomisations, the red line is the exponential fit to extract the gate fidelity F_{gate} and the grey area covers the standard deviation of the data. The error bar denotes the statistical error of the fit obtained through maximum likelihood estimation. The systematic error is expected to be on the order of $\sim 0.1\%$. We note that while sequence lengths of 100 Cliffords are sufficient to reach saturation for fidelities below 99%, longer sequences may be required to accurately resolve the fidelities of higher-performing qubits.

Supplementary Table S3. Offset and amplitude of measured Rabi oscillations.

	**Q1**	**Q2**	**Q3**	**Q4**	**Q5**	**Q6**	**Q7**	**Q8**	**Q9**	**Q10**
**A**	0.22	0.33	0.34	0.24	0.33	0.31	0.42	0.34	0.29	0.25
**B**	0.41	0.39	0.57	0.47	0.44	0.40	0.47	0.40	0.37	0.44

Supplementary Table S4. Fitting parameters of the RB datasets.

	**Q1**	**Q2**	**Q3**	**Q4**	**Q5**	**Q6**	**Q7**	**Q8**	**Q9**	**Q10**
**A**	0.197	0.323	0.353	0.203	0.336	0.371	0.407	0.357	0.366	0.305
**B**	0.436	0.385	0.529	0.382	0.504	0.444	0.465	0.424	0.474	0.488

Suppl. Note 3. QUBIT PROPERTIES IN THE INITIAL HOLE CONFIGURATION

Supplementary Table S5. Qubit properties in the original charge configuration at 41.4 mT.

label	Q1	Q2	Q3	Q4	Q5	Q6	Q7	Q8	Q9	Q10
**Charge occupation**	3	5	1	1	1	3	1	3	3	1
**Larmor frequency (MHz)**	302	293	319	347	361	321	355	335	327	331
**g-factor**	0.52	0.51	0.55	0.60	0.62	0.55	0.61	0.58	0.56	0.57
**coherence time T_2^* (ns)**	2381	2252	1710	2238	2041	2134	2319	2207	2018	1975
**Drive gate**	P1	P2	B5	B7	P5	P6	B12	P8	P9	B12
**Maximum driving strength (MHz/mV)**	0.15	0.19	0.05	0.06	0.08	0.21	0.13	0.15	0.09	0.11
**Single-qubit fidelity (%)**	99.9	99.6	99.5	99.6	99.6	99.8	99.6	99.7	99.5	99.6
**RB Rabi frequency** (MHz)	2.00	2.00	1.25	1.25	1.25	2.00	2.00	2.00	1.00	2.00

Suppl. Note 4. EXCHANGE INTERACTION IN THE 10-QUBIT ARRAY

Here we show the exchange interaction spectroscopy measurements for all ten qubits where we observe the exchange splitting between each qubit and one neighbouring qubit, see Figure **S3**. Figure **S4** shows qubit pairs, where we measured exchange interaction splitting for both qubits demonstrating the connectivity of the qubit array. The exchange interaction is probed as a function of virtual barrier gates that are defined in Suppl. Note 5.

Suppl. Note 5. GATE VIRTUALISATION

We use a set of virtual gates, defined in software, as outlined in Table **S6**. The first layer of virtualisation addresses crosstalk between gates and sensors as well as interactions between nearby quantum dots. This ensures that adjustments to the plunger or barrier gates do not shift the position of the charge sensor's Coulomb peak or change nearby dots potentials. In the second layer of virtualisation, the charging voltage of each dot is normalised by rescaling the strength of its corresponding plunger. Finally, the third layer virtualizes the barrier gates, enabling independent tuning of the exchange interaction between dots without changing their charge states. For a complete gate virtualisation method, readers are referred to Ref.[S4].

Supplementary Table S6. Table illustrating the layers of our virtualization approach. **S** denotes sensor plunger gates, **P** (**B**) defines quantum dot plunger (barrier) gates.

Virt. layer	Description	Notation
1	Charge sensor and QD compensations	$[\mathbf{vS}, \mathbf{vP}, \mathbf{vB}] = \mathbf{M_1} \cdot [\mathbf{S}, \mathbf{P}, \mathbf{B}]$
2	Normalisation of plungers with uniform charging voltages	$\mathbf{N} = \mathbf{M_2} \cdot \mathbf{vP}$
3	Barriers to QDs	$\mathbf{J} = \mathbf{M_3} \cdot [\mathbf{vB}, \mathbf{N}]$

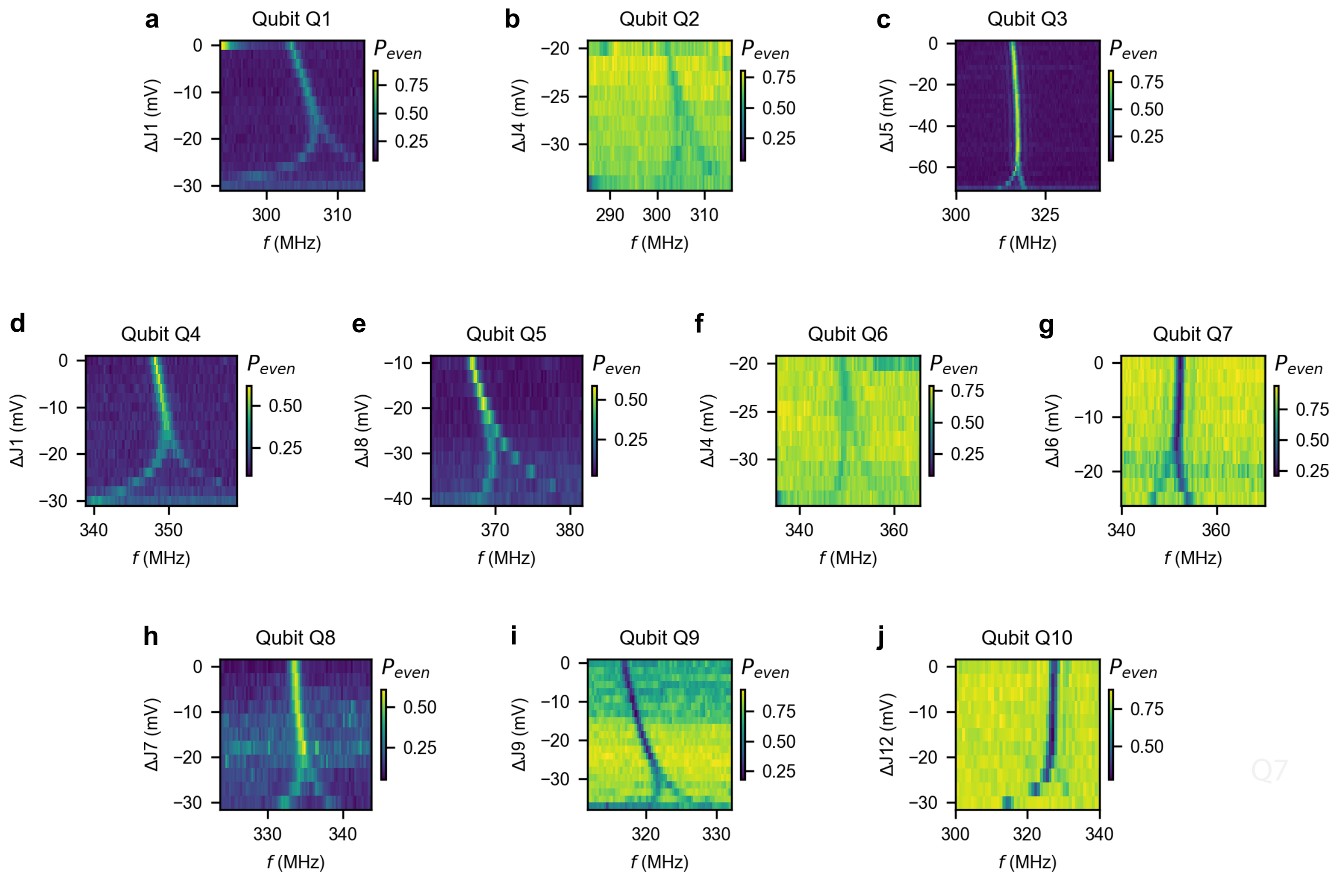

Supplementary Figure S3. **a-j. Exchange splitting for all ten qubits.** The observed splitting of the qubit resonance frequency as a function of virtual barrier voltage is directly proportional to the exchange coupling between qubits.

Suppl. Note 6. MODELING OF SINGLE- AND MULTI-HOLE QUANTUM DOTS

We have modeled the devices analytically and numerically to understand the trends highlighted by the experiments. We first introduce the structural models used in the simulations, then discuss the results for single-hole quantum dots. We finally derive an analytical model for the Rabi frequencies of multiply charged dots. This model, backed by numerical simulations, supports the enhancement of the Rabi frequencies in three-hole quantum dots.

A. Models and device

We consider the simplified geometry of Fig. **S5**, comprising a central plunger gate separated from its four nearest neighbors by the north-west (NW), north-east (NE), south-east (SE), and south-west (SW) barrier gates. Screening gates are also included between the NW/NE and SW/SE gate lines, to prevent, in particular, accumulation below the plunger gate line. The heterostructure and gate stack as well as the dimensions of the gates are borrowed from the experimental layout. This model geometry is close to the environment of dots Q5 and Q6.

We compute the potential in the heterostructure with a finite-volume Poisson solver, then the single-hole wave functions with finite-differences implementation of the four-band Luttinger-Kohn model [S5, S6], and finally the LSES and Rabi frequencies with the g-matrix formalism [S7–S9]. We also compute the three-hole and five-hole ground states and g-matrix with a full configuration interaction (FCI) method [S10]. Moreover, we construct a phenomenological theory that qualitatively captures the main features observed in the experiment by including only the few most relevant configurations of this FCI model.

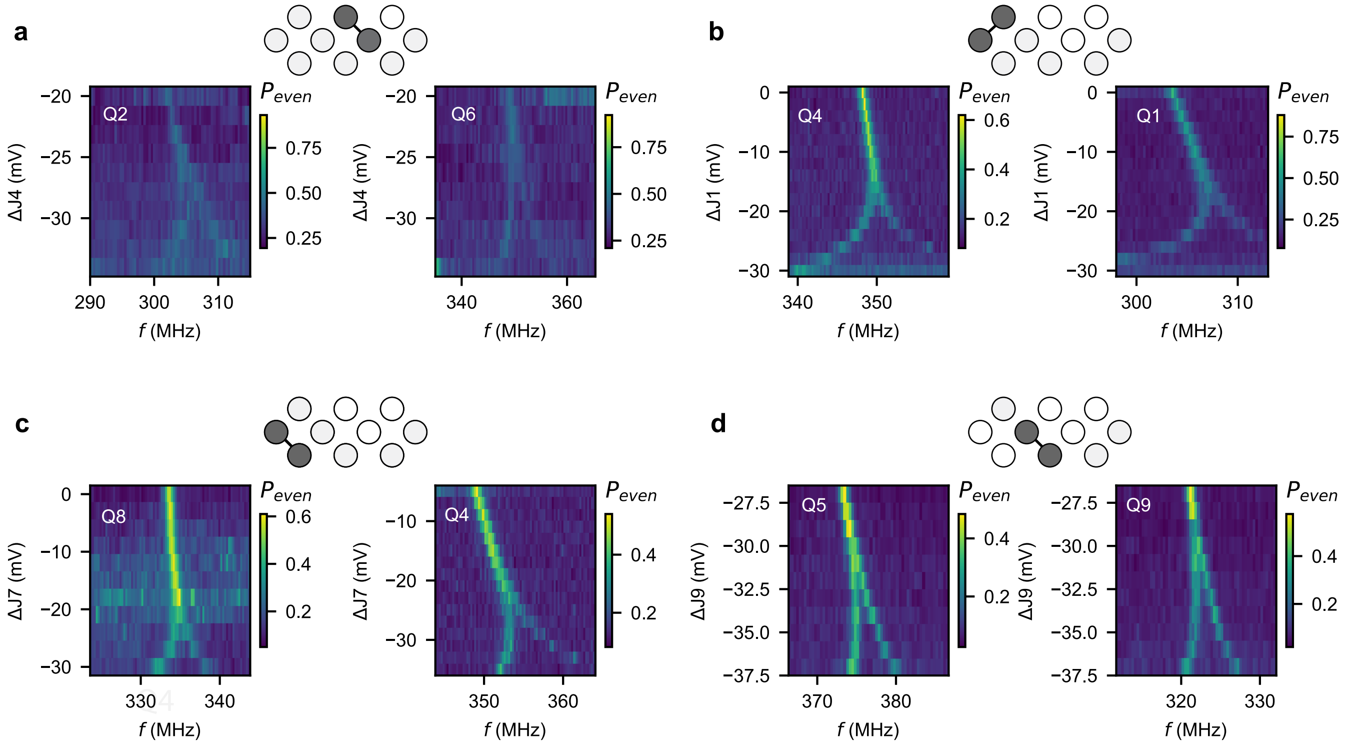

Supplementary Figure S4. a-d. Examplary data of exchange interaction between qubit pairs.

We add fixed trapped charges with density $\sigma = 5 \times 10^{11}\, e/\mathrm{cm}^2$ at the semiconductor/Al_2O_3 interface, either as a homogeneous sheet (which does not introduce disorder), or as a random distribution of point charges (see discussion below). We do not account here for the inhomogeneous strains imposed by the contraction of the metal gates upon cool-down [S11]. The results with such inhomogeneous strains are qualitatively similar; the Rabi frequencies with and without cool-down strains are broadly comparable at the experimental magnetic field orientation, while the average LSES of the barrier gates is typically smaller (resp. larger) than the experiment without (resp. with) these strains. This suggests that only part of the strains have been transferred to the heterostructure (due to, e.g., plasticity at the metal/oxide or oxide/semiconductor interfaces).

We first discuss the conclusions drawn from the modeling of single-hole dots, then of multiply charged dots.

B. Single-hole dots

We start from a bias point (Fig. **S6**a-c) where the ground-state hole wave function is well centered within the dot. The difference of potentials between the plunger and barrier gates ($\simeq 175\,\mathrm{mV}$) is similar to the experiment. We do not account for disorder at this stage (the distribution of charges at the semiconductor/Al_2O_3 interface is homogeneous). The dot is nonetheless slightly squeezed due to the asymmetry of the structure.

The g-tensor of the hole can be diagonalized in order to identify the principal g-factors g_X, g_Y and g_Z and the gyro-magnetic axes X, Y, Z; in this axis set, the effective g-factor g simply reads:

$$g^* = \sqrt{g_X^2 b_X^2 + g_Y^2 b_Y^2 + g_Z^2 b_Z^2}\,, \tag{3}$$

where $(b_X,\, b_Y,\, b_Z)$ are the coordinates of the unit vector oriented along the magnetic field $\vec{B}$ [S7, S12]. For a perfectly centered and symmetric dot, the gyro-magnetic axes coincide with the device x, y, z axes; in the present case $g_x^* \equiv |g_X| = 0.16$, $g_y^* \equiv |g_Y| = 0.043$ and $g_z^* \equiv |g_Z| = 13.46$. This strong anisotropy between in-plane and out-of-plane g-factors is characteristic of heavy-holes in Germanium. The imbalance between g_X and g_Y results from the slight squeezing of the dot [S9, S13]. For a magnetic field $\vec{B} = B(0,\, \sin\theta,\, \cos\theta)$ oriented 2.4° out of plane ($\theta = 92.4°$), the calculated effective g-factor $g^* = 0.56$ is dominated by the out-of-plane component g_Z.

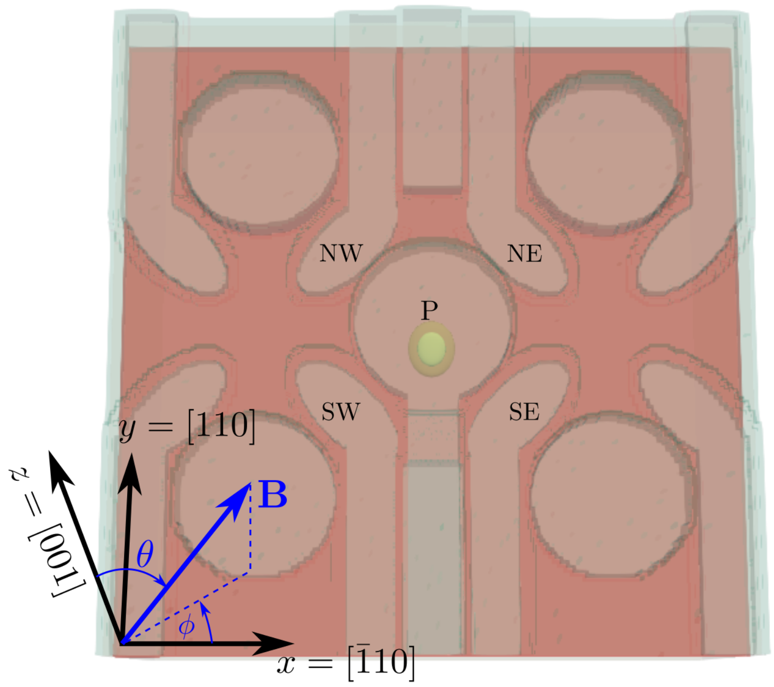

Supplementary Figure S5. The simulated device geometry, which includes the three layers of gates (barrier gates NW/NE/SW/SE, screening gates, and plunger gate P). The yellow shape below the plunger gate is the iso-density surface that encloses 80% of the charge of the "centered" dot of Fig. **S6**a-c. The orientation of the magnetic field $\vec{B}$ is characterized by the angles θ and ϕ in the crystallographic axes set $x = [\bar{1}10]$, $y = [110]$ and $z = [001]$.

The magnitudes of the LSES and Rabi frequencies computed at $B = 41\,\mathrm{mT}$ are reasonably comparable to the experiment given the uncertainties of the model (strains, exact nature and distribution of traps, Coulomb interactions between neighboring dots, ...). The LSES show generic features weakly dependent on the bias point and disorder:

- The LSES of the plunger gate is always positive and usually comparable or larger than the magnitude of the LSES of the barrier gates.
- The LSES of the barrier gates alternate positive and negative signs.

Indeed, raising the plunger gate voltage primarily deconfines the dot, which results in an increase of both in-plane and out-of-plane g-factors, thus in a strongly positive LSES [S9]. On the opposite, raising a barrier gate voltage further confines but deforms the dot, which results, in particular, in a decrease of g_Z. For such a symmetric dot, the LSES of all four barrier gates would actually be negative (blue) if these gates were only acting on the principal g-factors g_X, g_Y and g_Z. However, the barrier gates also tilt the gyro-magnetic X, Y, Z axes owing to the coupling between the in-plane and out-of-plane motions of the hole in the non-separable confinement potential of the dot (and owing to the inhomogeneous cool-down strains, when present) [S8, S11]. In particular, the gyro-magnetic Z axis rotates by a small angle $\delta\theta < 0$ when raising the SW and SE gate voltages. This brings the magnetic field closer to the effective equatorial (XY) plane, which decreases the contribution from the out-of-plane g-factor g_Z in Eq. (3) and further reduces the net g^*. On the opposite, raising the NW and NE gate voltages brings the magnetic field farther from the effective equatorial plane, which overcomes the decrease of g_Z and gives rise to a positive LSES.

The Rabi oscillations driven by the plunger gate essentially result from the modulations of the principal g-factors g_X, g_Y and g_Z (g-tensor modulation resonance or g-TMR). The plunger gate is, however, inefficient when the magnetic field is strictly in-plane [S8, S11]. Indeed, a disk-shaped quantum dot breathes homogeneously in the radio-frequency electric field of the plunger gate, which identically modulates $|g_X|$ and $|g_Y|$, and therefore does not act on the spin precession axis. This gives rise to a large LSES (as highlighted above), but to no transverse coupling (Rabi oscillations). The efficiency of the plunger gate however increases when the dot is significantly squeezed (because breathing is not isotropic in the XY plane any more) and/or when the magnetic field goes out of plane. On the contrary, the Rabi

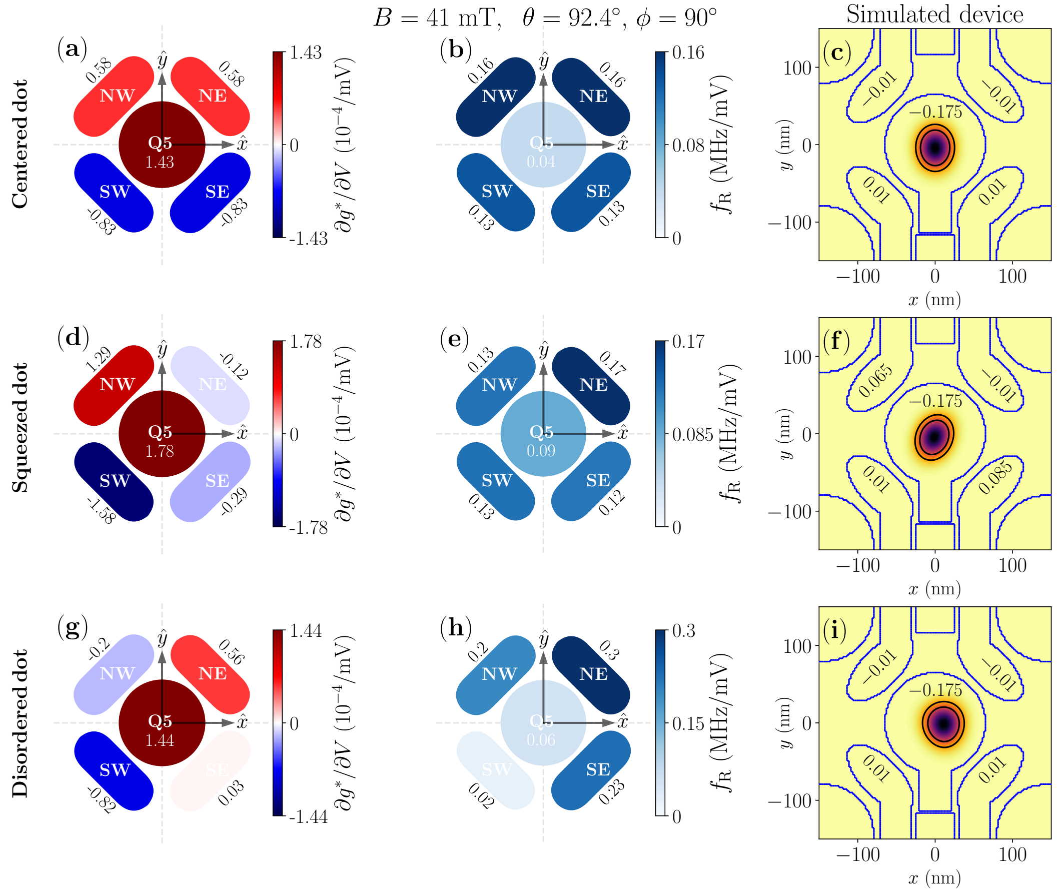

Supplementary Figure S6. (a-c) LSES, Rabi frequencies f_R and map of the squared wave function computed for a single hole in a "centered" dot. The LSES $\partial g/\partial V$ of the plunger and barrier gates (per mV) are reported in panel a), while the Rabi frequencies (in MHz/mV) are reported in panel b), and the bias voltages (in V) are reported in panel c). (d-f) Same for a "squeezed" dot at a different bias point. (g-i) Same for the displaced dot with charge disorder at the semiconductor/Al_2O_3 interface.

frequency of the barrier gates is dominated by the modulations of the gyromagnetic axes (also formally a g-TMR mechanism). They give rise to a $\propto \sigma_z$ drive term in the Hamiltonian [S8, S11] that is very efficient when the magnetic field is near plane.

The strength of these spin-orbit coupling mechanisms depends on the symmetry of the dot. The position and shape of the hole wave function can, in particular, be controlled by the barrier gates voltages. This is illustrated in Fig. **S6**d-f, where the bias has been tuned to squeeze the dot along the SW-NE axis. The in-plane gyro-magnetic axes tend to align with the main axes of this elliptical dot. The LSES of three out of the four barrier gates are now negative as a result of the new imbalance between the variations of the principal g-factors and the rotations of the gyro-magnetic (squeezing) axes. Yet the LSES of the plunger gate remains positive (whatever the position of the dot). Indeed, the dot is still essentially breathing when raising the plunger gate voltage, which increases all principal g-factors but hardly rotates the gyro-magnetic axes. The balance between the Rabi frequencies of the plunger and barrier gates is also impacted by the stronger asymmetry; in particular, the efficiency of the plunger gate is now comparable to the efficiency of the barrier gates (see above discussion).

Disorder may also change the symmetry of the hole wave function [S14]. It can have a strong impact on the sign of the LSES of the barrier gates, owing to the presence of "sweet lines" (zero LSES) of these gates near the equatorial plane of the unit sphere describing the magnetic field orientation [S9]. The disorder shuffles these sweet lines so that the magnetic field can practically end up on either side (positive or negative LSES). The effects of disorder are illustrated in Fig. **S6**g-i, for a particular distribution of positive point charges ($\sigma = 5 \times 10^{11}\, e/\mathrm{cm}^{-2}$) at the semiconductor/$Al_2O_3$ interface. We emphasize that this disorder is actually weak and has little incidence on the position and shape of the hole wave function (same bias point as in Fig. **S6**a-c). It has, nonetheless, sizable

impact on their derivatives (thus on the sign of the LSES and magnitude of the Rabi frequencies). Indeed, disorder does not only control the symmetry of the dot (together with the bias voltages), it also changes the response of the hole to electrical perturbations, by constraining the motion of the dot. This particular realization of disorder is in qualitative agreement with the experimental data for Q6 (color pattern of the LSES and Rabi frequencies); we can not claim however that Fig. **S6**i is a fair representation of the wave function of the singly-occupied Q6 as the matching bias/disorder is not unique for a single magnetic field orientation.

To conclude, we would like to comment on the impact of bias asymmetries and disorder on the dephasing time T_2^*. Assuming that the gates span a representative set of electrical perturbations, T_2^* is expected $\propto 1/\xi$ with $\xi = \sqrt{\sum_{\rm gate}(\partial g^*/\partial V_{\rm gate})^2}$ [S9]. The maps of ξ as a function of the magnetic field angles θ and ϕ are plotted in the main text (Fig. 3) for the device of Fig. **S6**g-i. Although the LSES of individual gates is strongly dependent on the orientation of the magnetic field, T_2^* shows much softer variations with the polar angle ϕ when the magnetic field goes out-of-plane [S9]. This results from the sum over gates (that is, the average over the orientation of the electric field fluctuations), and from the increasing contribution of the large g_Z to the effective g-factor g^*. Therefore, more robust (though not necessarily optimal) operations can be achieved with slightly out-of-plane magnetic fields.

C. Three and five-hole dots

We now address the three-hole and five-hole cases, starting with a discussion of the effects of Coulomb interactions on Rabi oscillations, and follow up with full configuration interaction (FCI) calculations.

The multi-spin system of many particles is exactly described by the Hamiltonian [S15]

$$\begin{aligned} H = &\sum_{\alpha}\left(\epsilon_\alpha\sigma_0 + \frac{1}{2}\mu_B\boldsymbol{\sigma}\cdot g_\alpha \mathbf{B}\right) \\ &+ \sum_{\alpha\neq\beta}\tau_{\alpha,\beta}^{s_\alpha,s_\beta} c_{\alpha,s_\alpha}^\dagger c_{\beta,s_\beta} \\ &+ \sum_{\alpha,\beta,\gamma,\delta}\Gamma_{\alpha,\beta,\gamma,\delta}^{s_\alpha,s_\beta,s_\gamma,s_\delta} c_{\alpha,s_\alpha}^\dagger c_{\beta,s_\beta} c_{\gamma,s_\gamma}^\dagger c_{\delta,s_\delta}\,, \end{aligned} \tag{4}$$

where σ_0 is the 2×2 identity matrix, and $\boldsymbol{\sigma} = (\sigma_x, \sigma_y, \sigma_z)^T$ is the Pauli-vector consisting of the three Pauli matrices. $\tau_{\alpha,\beta}$ are the standard inter-orbital tunneling elements between orbital α and β with spin $s_\alpha, s_\beta = \uparrow, \downarrow$ and $\Gamma_{\alpha,\beta,\gamma,\delta}$ are the Coulomb matrix elements connecting $\alpha,\beta,\gamma,\delta$ orbitals with spin $s_\alpha, s_\beta, s_\gamma, s_\delta = \uparrow,\downarrow$. The associated many-body wavefunctions can for example be constructed from single-particle eigenstates as described in Ref. [S15]. In that case, $\tau_{\alpha,\beta} = 0$ without perturbation.

Consequently, our system of interest, a single dot filled by 3 or 5 holes and orbitals $\alpha,\beta,\gamma,\delta = \{|0\rangle, |1\rangle, \cdots, |k\rangle\}$ is described by

$$H = \begin{pmatrix} \frac{1}{2}\mu_B\boldsymbol{\sigma}\cdot g_{E_0}\mathbf{B} + \sigma_0\mathcal{E}_{\mathrm{orb},E_0} & \sigma_0 t_{E_0,E_1} & \cdots & \sigma_0 t_{E_0,E_k} \\ \sigma_0 t_{E_1,E_0} & \frac{1}{2}\mu_B\boldsymbol{\sigma}\cdot g_{E_1}\mathbf{B} + \sigma_0\mathcal{E}_{\mathrm{orb},E_1} & \cdots & \vdots \\ \vdots & \vdots & \ddots & \vdots \\ \sigma_0 t_{E_k,E_0} & \cdots & \cdots & \frac{1}{2}\mu_B\boldsymbol{\sigma}\cdot g_{E_k}\mathbf{B} + \sigma_0\mathcal{E}_{\mathrm{orb},E_k} \end{pmatrix}. \tag{5}$$

Here, $|E_0\rangle$ describes a configuration where the ground state orbital $|0\rangle$ is doubly occupied and the remaining hole occupies the lowest excited orbital $|1\rangle$. The states $|E_k\rangle$ with $k > 0$ are excited configurations. Considering only single excitations, the tunnel matrix elements are given by $t_{\alpha,\beta} = \tau_{\alpha,\beta} + \sum_\gamma(\Gamma_{\alpha,\beta,\gamma,\gamma} + \Gamma_{\gamma,\gamma,\alpha,\beta} + \Gamma_{\alpha,\gamma,\gamma,\beta} + \Gamma_{\gamma,\beta,\alpha,\gamma})$, where the sum γ goes over all occupied orbitals. Similarly, the orbital energies are given by $\mathcal{E}_{\mathrm{orb},\alpha} = \epsilon_\alpha + \sum_\gamma(\epsilon_\gamma + \Gamma_{\alpha,\alpha,\gamma,\gamma} + \Gamma_{\gamma,\gamma,\alpha,\alpha} + \Gamma_{\alpha,\gamma,\gamma,\alpha} + \Gamma_{\gamma,\alpha,\alpha,\gamma})$. Note that these terms can include both non-interacting single-particle terms and many-body contributions.

Here, we neglect the spin-orbit interactions between close quantum dots and between different orbitals, which are usually small for quasi-circular dots in compressively strained planar Germanium channels. However, in general, one can include the spin-orbit contributions to all inter-orbital transition matrix elements $\sigma_0 t_{\alpha,\beta} \to \cos(\zeta_{\alpha,\beta})\sigma_0 t_{\alpha,\beta} + \sin(\zeta_{\alpha,\beta})\boldsymbol{\sigma}\cdot\mathbf{t}_{\mathrm{soi},\alpha,\beta}$ and orbital energies $\sigma_0\mathcal{E}_{\mathrm{orb},\alpha} \to \cos(\zeta_\alpha)\sigma_0\mathcal{E}_{\mathrm{orb},\alpha} + \sin(\zeta_\alpha)\boldsymbol{\sigma}\cdot\mathcal{E}_{\mathrm{orb,soi},\alpha}$.

With this assumptions, we can capture the main experimental features, such as the altered Rabi frequency in the three-hole dot. As we will show by numerical simulations in the next section, this is because in the regime explored in our experiment, the dominant change of the Rabi frequency is caused by a non-interacting contribution emerging from

the lower symmetry of the p-type wavefunctions of the higher orbital states. The many-body correction quantitatively renormalizes this contribution. Below, we show that our analytical model captures the main features of the single-body and many-body contributions, thus it constitutes an appropriate tool to interpret multi-hole quantum dots.

1. Analytical expressions

To find approximate analytical expressions we only consider two Slater orbitals $\{|E_0\rangle, |E_1\rangle\}$, i.e. we now assume $|t_{E_0,E_1}|/(\mathcal{E}_{\mathrm{orb},E_1} - \mathcal{E}_{\mathrm{orb},E_0}) \gg |t_{E_0,E_k}|/(\mathcal{E}_{\mathrm{orb},E_k} - \mathcal{E}_{\mathrm{orb},E_0})|$, which can be expected for two-fold quasi-degeneracy, e.g. p-orbitals of the 2D harmonic oscillator. This simplification allows us to construct a simple yet meaningful analytical theory, which is analogous to that of a flopping-mode spin qubit. With this theory, we can qualitatively interpret observed experimental features. We note that the case of multiple strongly coupled orbitals can be treated similarly. To be more quantitative, we perform FCI simulations of the system in the next section. These numerical simulations also extend to squeezed quantum dots [S13], similar to that shown for a single hole in Fig. **S6**d-f. Even for such squeezed dots, we find a good qualitative match with this simple, effective model.

The ground-states are given by block-diagonalizing the lowest two levels of Hamiltonian (5). Up to energy shifts, the ground-state Hamiltonian is well-approximated by

$$H_{\mathrm{eff}} \approx \frac{\mu_B}{2}\left[\boldsymbol{\sigma}\cdot g_{E_0}\mathbf{B} + \boldsymbol{\sigma}\cdot g_{E_1}\mathbf{B} + \cos(\zeta)\left(\boldsymbol{\sigma}\cdot g_{E_0}\mathbf{B} - \boldsymbol{\sigma}\cdot g_{E_1}\mathbf{B}\right)\right], \tag{6}$$

with $\zeta = \arctan(\mathcal{E}_{\mathrm{orb},E_1} - \mathcal{E}_{\mathrm{orb},E_0}, 2t_{E_0,E_1})$. The intra-dot interaction hybridizes the spin and orbital degrees similarly to multi-dot spin-charge qubits such as the flopping-mode qubit. Consequently, we expect that the interacting hole system could behave similarly and a simple flopping-mode Hamiltonian could phenomenologically describe the enhancement of Rabi frequencies observed in the experiment.

The Rabi frequency from driving gate $V^{(k)} \to V^{(k)} + V_{\mathrm{ac}}^{(k)}$ is given by

$$f_R = \frac{\mu_B V_{\mathrm{ac}}^{(k)}}{2h}\frac{||(g\mathbf{B}) \times (\frac{dg}{dV}\mathbf{B})||}{||g\mathbf{B}||}. \tag{7}$$

Here the total g-tensor is given by

$$g = \frac{1}{2}\left[g_{E_0} + g_{E_1} + \cos(\zeta)\left(g_{E_0} - g_{E_1}\right)\right] \tag{8}$$

and the derivative with respect to gate voltages are:

$$\frac{dg}{dV^{(k)}} = \cos^2\left(\frac{\zeta}{2}\right)\frac{dg_{E_0}}{dV^{(k)}} + \sin^2\left(\frac{\zeta}{2}\right)\frac{dg_{E_1}}{dV^{(k)}} + \frac{1}{2}\left(g_{E_0} - g_{E_1}\right)\frac{d\cos(\zeta)}{dV^{(k)}} \tag{9}$$

$$\frac{d\cos(\zeta)}{dV^{(k)}} = 2\,\frac{t_{E_0,E_1}\left(\frac{d}{dV^{(k)}}\mathcal{E}_{\mathrm{orb},E_1} - \frac{d}{dV^{(k)}}\mathcal{E}_{\mathrm{orb},E_0}\right) + \left(\mathcal{E}_{\mathrm{orb},E_1} - \mathcal{E}_{\mathrm{orb},E_0}\right)\frac{d}{dV^{(k)}}t_{E_0,E_1}}{(\mathcal{E}_{\mathrm{orb},E_1} - \mathcal{E}_{\mathrm{orb},E_0})^2 + t_{E_0,E_1}^2}. \tag{10}$$

The Rabi frequency thus has two important contributions: A conventional g-tensor contribution arising from the sum and differences of the two individual g-tensors and a novel contribution including the many-body interactions that provides a similar enhancement in driving efficiency as the flopping mode qubit.

a. Static g-tensor modification The conventional g-tensor contributions are modified from the single-hole case due to the frozen core that occupies the ground-state. In the case of 3 holes, the quasi-degenerate ground states can then be well approximated by the superpositions of $|1_{x'}, 0_{y'}\rangle$ and $|0_{x'}, 1_{y'}\rangle$

$$|E_0\rangle = \cos(\theta_p)\,|1_{x'}, 0_{y'}\rangle + \sin(\theta_p)\,|0_{x'}, 1_{y'}\rangle\,, \tag{11}$$

$$|E_1\rangle = \cos(\theta_p)\,|0_{x'}, 1_{y'}\rangle - \sin(\theta_p)\,|1_{x'}, 0_{y'}\rangle\,, \tag{12}$$

where $(x', y') = ([100], [010])$ is a coordinate system rotated by 45°, $|n_{x'}, m_{y'}\rangle$ is short-hand notation for n-th and m-th orbital in (x', y') direction, and $\theta_p = \theta_p(V^{(k)})$ is the in-plane angle pointing towards weaker confinement which depends on the applied voltages. Analogously, in the case of 5 holes, the quasi-degenerate ground states can be roughly approximated by the superpositions of $|1_{x'}, 0_{y'}\rangle$, $|0_{x'}, 1_{y'}\rangle$, $|2_{x'}, 0_{y'}\rangle$, and $|0_{x'}, 2_{y'}\rangle$

$$|E_0\rangle = \cos(\theta_p)\,|0_{x'}, 1_{y'}\rangle - \sin(\theta_p)\,|1_{x'}, 0_{y'}\rangle\,, \tag{13}$$

$$|E_1\rangle = \cos(\theta_p)\,|2_{x'}, 0_{y'}\rangle + \sin(\theta_p)\,|0_{x'}, 2_{y'}\rangle\,. \tag{14}$$

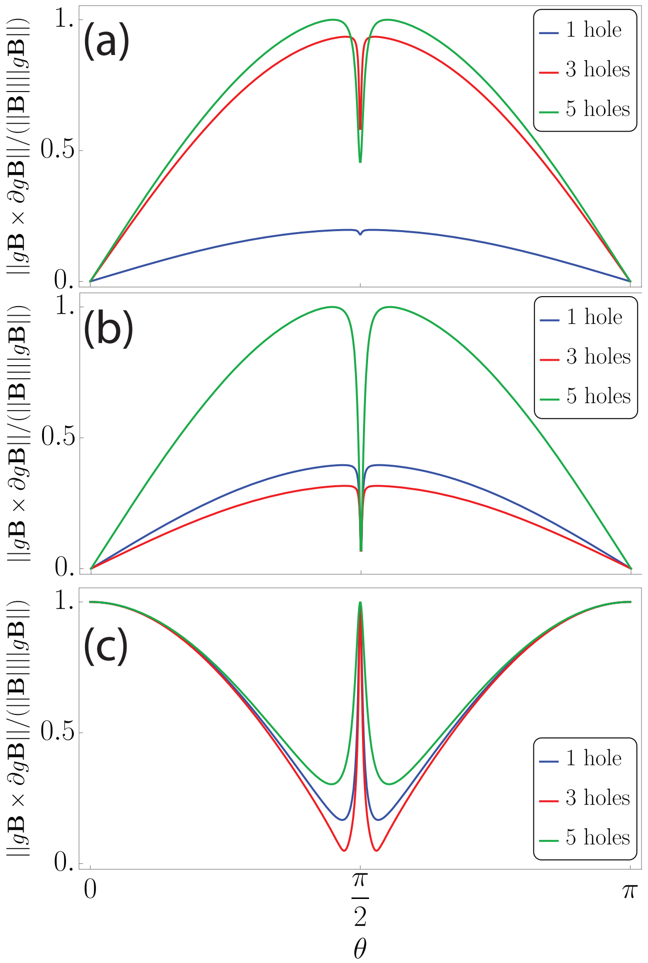

Supplementary Figure S7. Dependence of the driving prefactor $||g_{E_0}\mathbf{B} \times \partial g_{E_0}\mathbf{B}||/(B||g\mathbf{B}||)$ considering (a) $\partial g_{E_0} \propto \frac{dg_{E_0}}{d\langle p^2\rangle}$, (b) $\partial g_{E_0} \propto \frac{dg_{E_0}}{d\gamma_h}$, and (c) $\partial g_{E_0} \propto \begin{pmatrix} 0 & 0 & 1 \\ 0 & 0 & 1 \\ 1 & 1 & 0 \end{pmatrix}$ on the magnetic field angle θ for magnetic field angle $\phi = 90^\circ$ in arbitrary units. For the single holes case, the hole occupies the ground-state s-orbital of a rotated asymmetric 2D harmonic oscillator. For the 3 holes case, two holes fill the ground-state s-orbital and the remaining hole occupies the lower p-orbital of a rotated asymmetric 2D harmonic oscillator that corresponds to the $|E_0\rangle$ in Eq. (11) with $\theta_p = 65^\circ$. For the 5 holes case, two holes fill the ground-state s-orbital and two holes fill the lower p-orbital and the remaining hole occupies the higher p-orbital and corresponds to the $|E_0\rangle$ state in Eq. (13) with $\theta_p = 65^\circ$. For all calculations, we used approximate expressions from Eqs. (15)-(19) and utilize the relation $\langle p_\xi^2\rangle_n = (2n+1)\langle p_\xi^2\rangle_0$, where n is the n-th excited harmonic oscillator state in weak and strong confinement directions. Furthermore, we used for the Bohr radius along the weak and strong confinement axis $L_{\text{weak}} = 27\,\text{nm}$ and $L_{\text{strong}} = 21.5\,\text{nm}$, $\gamma_h = 3.9$, $\lambda = 9.4$, $\lambda' = 18.2$, and $\widetilde{\lambda} = 16.4$ estimated from fitting to numerical simulations of the 1 hole wavefunction.

We note, however, that for the 5 hole case, there is another close orbital state, where a hole from the frozen lower p-orbital is excited and freezes the higher p-orbital. This gives rise to a similar situation as for the 3 hole case with ground and excited state interchanged.

The total g-tensor neglecting strain is then approximately given by [S11, S16, S17]

$$g_{zz} \approx 6\kappa + \frac{27}{2}q - 2\gamma_h \tag{15}$$

$$g_{xx} \approx 3q - \frac{6(\lambda \langle E_{0,1}| p_{x'}^2 |E_{0,1}\rangle - \lambda' \langle E_{0,1}| p_{y'}^2 |E_{0,1}\rangle)}{m_0 \Delta_{\mathrm{HL}}} \tag{16}$$

$$g_{yy} \approx -3q + \frac{6(\lambda \langle E_{0,1}| p_{y'}^2 |E_{0,1}\rangle - \lambda' \langle E_{0,1}| p_{x'}^2 |E_{0,1}\rangle)}{m_0 \Delta_{\mathrm{HL}}}, \tag{17}$$

$$g_{xy,yx} \approx \mp \frac{12\widetilde{\lambda} \langle E_{0,1}| p_{x'} p_{y'} |E_{0,1}\rangle}{m_0 \Delta_{\mathrm{HL}}}, \tag{18}$$

$$g_{xz,yz,zx,zy} \approx 0, \tag{19}$$

where $\kappa \approx 3.4$ and $q = 0.06$ are material parameters, γ_h is the out-of-plane g-factor renormalization due to confinement, the dimensionless parameters λ, λ', and $\widetilde{\lambda}$ are combinations of transition matrix elements between out-of-plane wavefunctions explicitly defined in Ref. [S17], and Δ_{HL} is the heavy-hole light-hole splitting. The Rabi frequency from g-tensor modulation is then given by [S8]

$$\mathbf{f}_R^{E0} \equiv \frac{\mu_B V_{\mathrm{ac}}^{(k)}}{2h||g\mathbf{B}||} (g_{E_0}\mathbf{B}) \times \left(\frac{dg_{E_0}}{dV}\mathbf{B} \right). \tag{20}$$

To better illustrate the effects emerging in the three-hole quantum dots when driving with the plunger gates, we plot in Fig. **S7** the dimensionless prefactor $||g_{E_0}\mathbf{B} \times \partial g_{E_0}\mathbf{B}||/(B||g_{E_0}\mathbf{B}||)$ considering the conventional g-tensor modulation resonance via (a) plunger gate driving $\partial g_{E_0} \propto \frac{dg_{E_0}}{d\langle p^2\rangle}$ and (b) $\partial g_{E_0} \propto \gamma_h$ modulation, and (c) other spin-orbit interaction mechanisms [S8, S13, S18, S19] $\partial g_{E_0} \propto \begin{pmatrix} 0 & 0 & 1 \\ 0 & 0 & 1 \\ 1 & 1 & 0 \end{pmatrix}$ as a function of the out-of-plane magnetic field angle θ ($\phi = 90°$). We use Eqs. (15)-(19) to compute g_{E_0}. As seen in Fig. **S7** (a), modulating $\langle p^2\rangle$ changes the diagonal g-tensor coefficients, in particular their relative amplitude, giving rise to a "breathing" modulation, which in combination with out-of-plane magnetic fields leads to a large Rabi frequency for the multi-hole case. We observe that the plunger gate contribution is small (but not zero) for exactly in-plane magnetic fields, maximal at a magic angle, and vanishes at $\theta = 0°$. We notice in Fig. **S7** (b) a similar dependence with respect to θ for driving γ_h. Here, we see that the 3 hole case is similar to the single hole case. The main difference is that in this case, we do not activate the "breathing" mode. As shown by Fig. **S7** (c), the spin-orbit interaction, typically induced by barrier gate modulations, which leads to the traditional driving mechanisms for single-hole spins [S8, S13, S18, S19] is efficient at exactly $\theta = 90°$ and close to $\theta = 0°$. The numerical simulations include all these terms, that are of practical relevance and lead to efficient driving for out-of-plane magnetic field directions.

b. Many-body contribution The many-body contribution can be explicitly expressed by

$$\mathbf{f}_R^{MB} \equiv \frac{\mu_B V_{\mathrm{ac}}^{(k)}}{8h||g\mathbf{B}||} \left[g_{E_0} + g_{E_1} + \cos(\zeta)\,(g_{E_0} - g_{E_1})\right] \mathbf{B} \times \left[\frac{d\cos(\zeta)}{dV^{(k)}}\,(g_{E_0} - g_{E_1}) \right] \mathbf{B} \tag{21}$$

$$= -\frac{\mu_B V_{\mathrm{ac}}^{(k)}}{4h||g\mathbf{B}||} \frac{d\cos(\zeta)}{dV^{(k)}} g_{E_0}\mathbf{B} \times g_{E_1}\mathbf{B}. \tag{22}$$

The tunneling between different eigenstates, that include the Coulomb matrix elements and orbital energies, are strongly affected by deformations (breathing) and less by lateral movement. Firstly, this implies that the top plunger gate can potentially lead to a larger Rabi frequency than barrier or other plunger gates. Secondly, nearby barrier gates can also lead to larger Rabi frequencies, as they also deform the wave-functions. Lastly, we expect the contribution to the Rabi frequency from distant gates to rapidly drop, since they dominantly shift the wave-function laterally.

To better illustrate the additional effects emerging in the three-hole quantum dots, we plot in Fig. **S8** the prefactor $||g_{E_0}\mathbf{B} \times g_{E_1}\mathbf{B}||/(B||g\mathbf{B}||)$ of the many-body contribution as a function of the out-of-plane magnetic field angle θ ($\phi = 90°$). We consider the same settings as before and use Eqs. (15)-(19) to compute g_{E_0} and g_{E_1}. We observe that the many-body contribution is small (but not zero) for exactly in-plane magnetic fields, maximal at the magic angle, and vanishes at $\theta = 0°$ following a similar trend than Fig. **S7**(a).

This many-body contribution adds to the conventional single-particle g-factor modulations. We emphasize as shown above that the conventional contribution also differs from the single-hole case as the symmetry of the occupied orbitals is not the same. The conventional contribution also experiences an enhancement that depends on the elongation of the orbitals and on the magnetic field direction [S13]. While in this experiment the total Rabi frequency is enhanced,

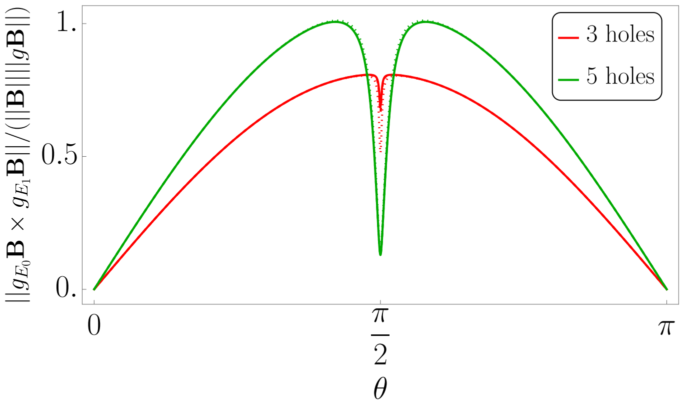

Supplementary Figure S8. Dependence of the driving prefactor $||g_{E_0}\mathbf{B} \times g_{E_1}\mathbf{B}||/(B||g\mathbf{B}||)$ on the magnetic field angle θ for 3 (red) and 5 (green) holes and $\phi = 90°$. For the 3 holes case, two holes fill the ground-state s-orbital and the remaining hole occupies the lower p-orbital of a rotated asymmetric 2D harmonic oscillator that corresponds to the $|E_0\rangle$ in Eq. (11) with $\theta_p = 65°$. The closest excited state is the higher p-orbital of a rotated asymmetric 2D harmonic oscillator that corresponds to the $|E_1\rangle$ in Eq. (12) with $\theta_p = 65°$. For the 5 holes case, two holes fill the ground-state s-orbital and two holes fill the lower p-orbital and the remaining hole occupies the higher p-orbital and corresponds to the $|E_0\rangle$ state in Eq. (13) with $\theta_p = 65°$. The closest excited state is the lowest d-orbital of a rotated asymmetric 2D harmonic oscillator that corresponds to the $|E_1\rangle$ state in Eq. (14) with $\theta_p = 65°$. Solid, dashed, dotted lines correspond to a mixing angle $\zeta = 1°, 10°, 30°$. The dashed line overlaps with the solid line and is hardly visible. The dotted line shows clear deviation close to $\theta = \pi/2$. For this calculation, we used approximate expressions from Eqs. (15)-(19) and utilize the relation $\langle p_\xi^2 \rangle_n = (2n+1) \langle p_\xi^2 \rangle_0$, where n is the n-th excited harmonic oscillator state in $\xi, \chi = x', y'$ directions. Furthermore, we used for the Bohr radius along the weak and strong confinement axis $L_{\mathrm{weak}} = 27\,\mathrm{nm}$ and $L_{\mathrm{strong}} = 21.5\,\mathrm{nm}$, $\gamma_h = 3.9$, $\lambda = 9.4$, $\lambda' = 18.2$, and $\widetilde{\lambda} = 16.4$ estimated from fitting to numerical simulations of the 1 hole wavefunction.

we stress that in general these two contributions can interfere constructively or destructively, and thus we cannot exclude a priori particular scenarios where the three-hole Rabi frequencies decrease compared to non-interacting holes. This highlights the complexity of the spin dynamics in the many-particle case. We provide an illustration in the next section using configuration interaction simulations that account for all contributions and their relative interplay.

2. Numerical simulations

We discuss the main features of the LSES and Rabi frequencies in three and five-hole quantum dots and illustrate the role of Coulomb interactions with numerical simulations.

The LSES, Rabi frequencies and wave functions of a single, three and five-hole dot similar to Fig. **S6**d-f are shown in Fig. **S9**. They are computed in the non-interacting limit. Therefore, the LSES and Rabi frequencies are those, respectively, of the ground, first, and second excited Kramers pair. The latter two are the p-like orbitals displayed in Fig. **S9**f and **S9**i. They are effectively split by the bias asymmetries (here, by $\Delta E = 1.064\,\mathrm{meV}$), enabling the formation of well-defined qubits. Remarkably, the LSES of the plunger gate is negative in the three and five-hole dots, while the Rabi frequency of the plunger gate is much larger in the three than in the single and five-hole dots. As discussed before, raising the plunger gate voltage results in an increase of all principal g-factors in the single hole case, which gives rise to a large LSES but to little transverse coupling. The asymmetry of the split p-like orbitals and their inhomogeneous response to the gate field lead to much more anisotropic modulations of the gyromagnetic factors. In particular, the in-plane and out-of-plane g-factors vary in opposite ways when driving the plunger gate, enabling efficient g-TMR for slightly off-plane magnetic fields. The Rabi frequency decreases in the five-hole dots because the second p-orbital is more confined along its main axis (and thus less responsive to the plunger gate). This scenario generally holds as long as the symmetry is broken and the two p-orbitals are reasonably split. Notably, driving the barrier gates can mix the two p-orbitals, which results in rotations of their axes and to modulations of the g-tensor elements g_{xy} and g_{yx} [S8].

We have performed FCI simulations to assess the effects of Coulomb interactions in the three-hole case [S20]. We diagonalize exactly the many-body Hamiltonian [Eq. (5)] in a basis of all Slater determinants built from the first 48 single-particle orbitals computed on the finite-differences grid (Sec. Suppl. Note 6 B) [S10]. The LSES and Rabi frequencies of each gate, as well as the map of the three-hole density are plotted in Fig. **S10**. At variance with the squared wave functions of Fig. **S9**, the density includes the contributions from the three holes. It shows a dip at

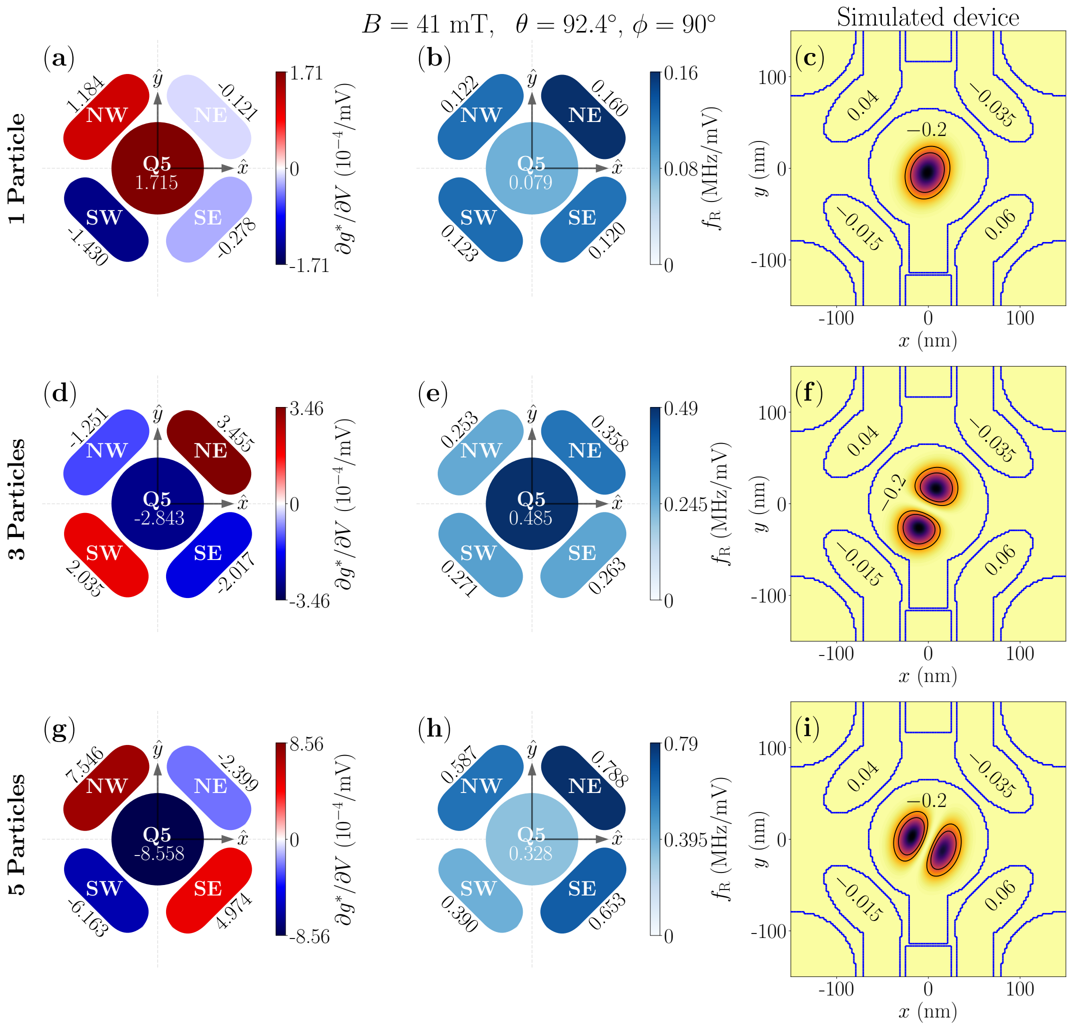

Supplementary Figure S9. (a-c) LSES, Rabi frequencies f_R and map of the squared wave function computed for the ground Kramers pair in a "squeezed" dot similar to Fig. **S6**d-f. The LSES $\partial g/\partial V$ of the plunger and barrier gates (per mV) are reported in panel a), while the Rabi frequencies (in MHz/mV) are reported in panel b), and the bias voltages (in V) are reported in panel c). (d-f) Same for the first excited Kramers pair (three non-interacting holes). (g-i) Same for the second excited Kramers pair (five non-interacting holes).

the center reminiscent of the p-like character of the second Kramers pair. The configuration with two holes in the ground s-orbital and one hole in this p-orbital accounts for 63% of the many-body wave function. The dot is more extended along the major axis than in Fig. **S9**f, because the Coulomb interactions tend to separate the holes. The splitting between the ground and first excited doublets is reduced from $\Delta E = 1.064\,\mathrm{meV}$ (non-interacting limit) to $\Delta E = 0.784\,\mathrm{meV}$ by the interactions. The Coulomb correlations thus leave visible fingerprints in the spectrum and wave functions of the dots. Yet they are not large enough to destabililize the dots and rule the physics of the device (with, e.g., the emergence of Wigner localization effects [S10]). Germanium quantum dots are, indeed, expected to be more resilient to Coulomb interactions than silicon quantum dots because of their smaller valence band effective masses. Nevertheless, the significant mixing with other orbital configurations leads to quantitative changes in the gyromagnetic response. On the one hand, the sign of the LSES of the plunger gate and of two barrier gates has changed (with respect to the non-interacting limit). The LSES of the plunger gate is thus now positive as in the experiments. As discussed for the non-interacting case, the sign of the LSES is very sensitive to electrical perturbations (including interactions) when the magnetic field lies close to the equatorial plane due to the presence of nearby sweet lines (zero LSES lines) that move when the interactions are switched on. The interactions have, on the other hand, much less impact on the Rabi frequencies, whose gradients along these sweet lines are usually small due to "reciprocal sweetness" considerations [S10]. The Rabi frequencies are actually smaller than in the non-interacting limit, but this shall not

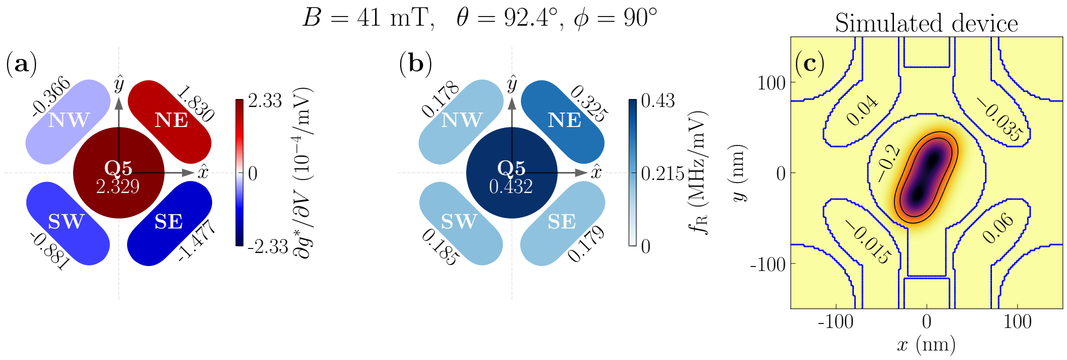

Supplementary Figure S10. (a) LSES, (b) Rabi frequencies f_{R} and (c) map of the density computed for three interacting holes in the same dot as in to Fig. **S9**.

be interpreted as a screening of the drive by the interacting holes (which is higher-order in the drive amplitude). This results, as discussed above, from the destructive interference between the different configurations mixed in the CI wave function, which could possibly be constructive in different conditions. The relative driving efficiencies of the gates is similar to Fig. **S9**e. The plunger gate remains, in particular, more efficient than the barrier gates in the interacting three-hole regime. As in the non-interacting limit, the Rabi oscillations driven by the plunger gate essentially result from the opposite modulations of the in- and out-of-plane g-factors.

Calculations in interacting five-hole dots are much more expensive and difficult to converge. The results with Slater determinants built from the first 32 single-particle orbitals, although not as well converged, draw a scenario similar to the three-hole case. In particular, the Rabi frequencies of the different gates follow the same trends as in Fig. **S9**h, but are significantly smaller than in the non-interacting case (down to $f_R \approx 0.16\,\mathrm{MHz/mV}$ for the plunger gate). This decrease of the efficiency of the plunger gate from three to five holes is in line with the experimental results.

To conclude, our analytical and numerical models reproduce and explain many generic features of the experimental data, including the sign of the LSES of the plunger gate, the versatility of the color patterns of the LSES of the barrier gates, and the enhancement of the Rabi frequency of the plunger gate in three-hole dots. It provides insights into the physics at work in these devices. It shows, in particular, that the LSES and Rabi oscillations result from a combination of g-TMR mechanisms involving modulations of the principal g-factors as well as rotations of the gyro-magnetic axes. The strength of these mechanisms is dependent on the symmetry of the hole wave function, thus on the dot occupation, barrier gate voltages and level of disorder. The Coulomb correlations leave visible fingerprints on the gyromagnetic response of the dots (in particular, on the sign of the LSES and on the magnitude of the Rabi frequencies), but the main qualitative trends and physics can still be understood from the non-interacting limit. The robustness of the physics of Germanium quantum dots with respect to Coulomb interactions, favored by small valence band effective masses, is actually a strength for the development of reliable and scalable spin qubit technologies.

Suppl. Note 7. EDSR DRIVING EFFICIENCY

Additional data of EDSR driving efficiency as a function of driving gate and charge occupation are shown in Figure **S11**.

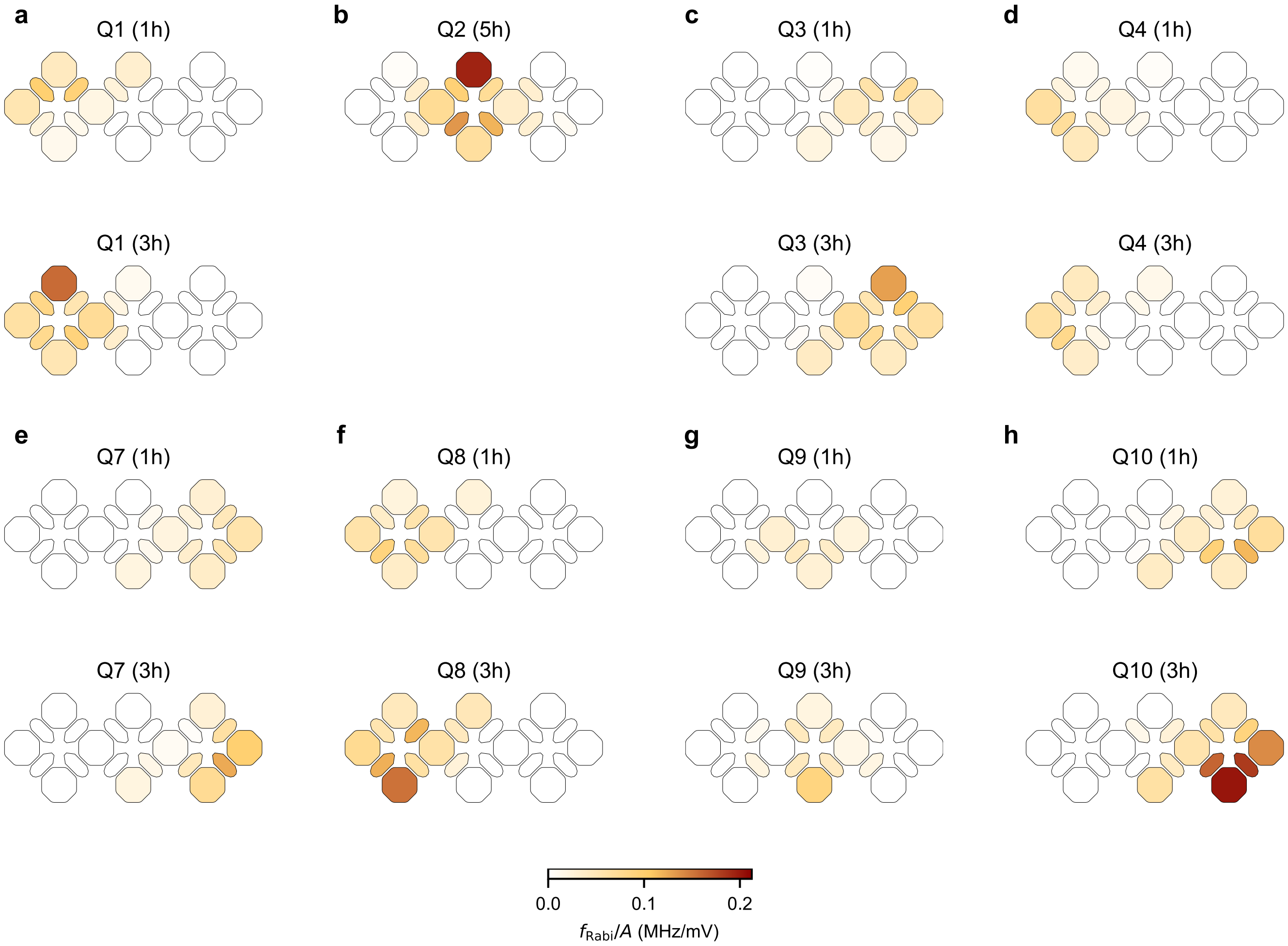

Supplementary Figure S11. Extended data of the EDSR driving efficiency for Q1 (a), Q2 (b), Q3 (c), Q4 (d), Q7 (e), Q8 (f), Q9 (g), Q10 (h) in the single- and three- hole configuration for all qubits except Q2 which is only probed in the five-hole occupancy.

Suppl. Note 8. G-FACTOR TUNABILITY

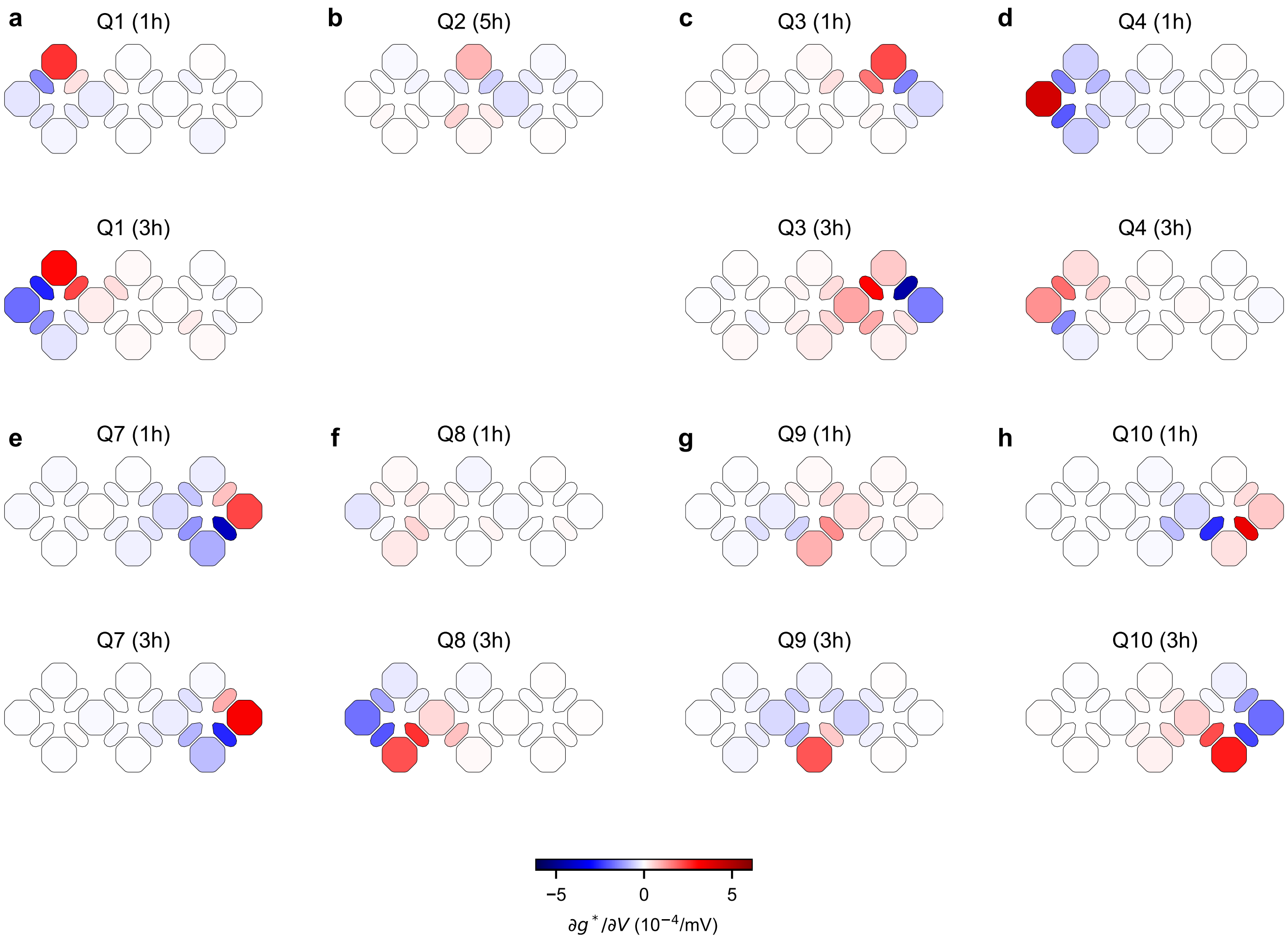

Supplementary Figure S12. Extended data of the g-factor tunability for Q1 (a), Q2 (b), Q3 (c), Q4 (d), Q7 (e), Q8 (f), Q9 (g), Q10 (h) in the single- and three- hole configuration for all qubits except Q2 which is only probed in the five-hole occupancy. More details about data analysis can be found in Suppl. Note S10.

Suppl. Note 9. LSES EXTRACTION

To extract the g-factor susceptibility of a qubit to each gate, microwave frequency sweeps around the Larmor frequency have been performed, while changing the applied voltage on each gate one after the other. In this experiment, we used a chirp signal to probe the resonance frequency of each qubit. After, the resonance frequency for each configuration is determined and fitted with a linear fit. The corresponding slope indicates the gate susceptibility $\partial f_{\mathrm{Rabi}}/\partial V_{\mathrm{gate}}$. In Figure **S13** and **S14** examples of the data and the corresponding linear fits are plotted.

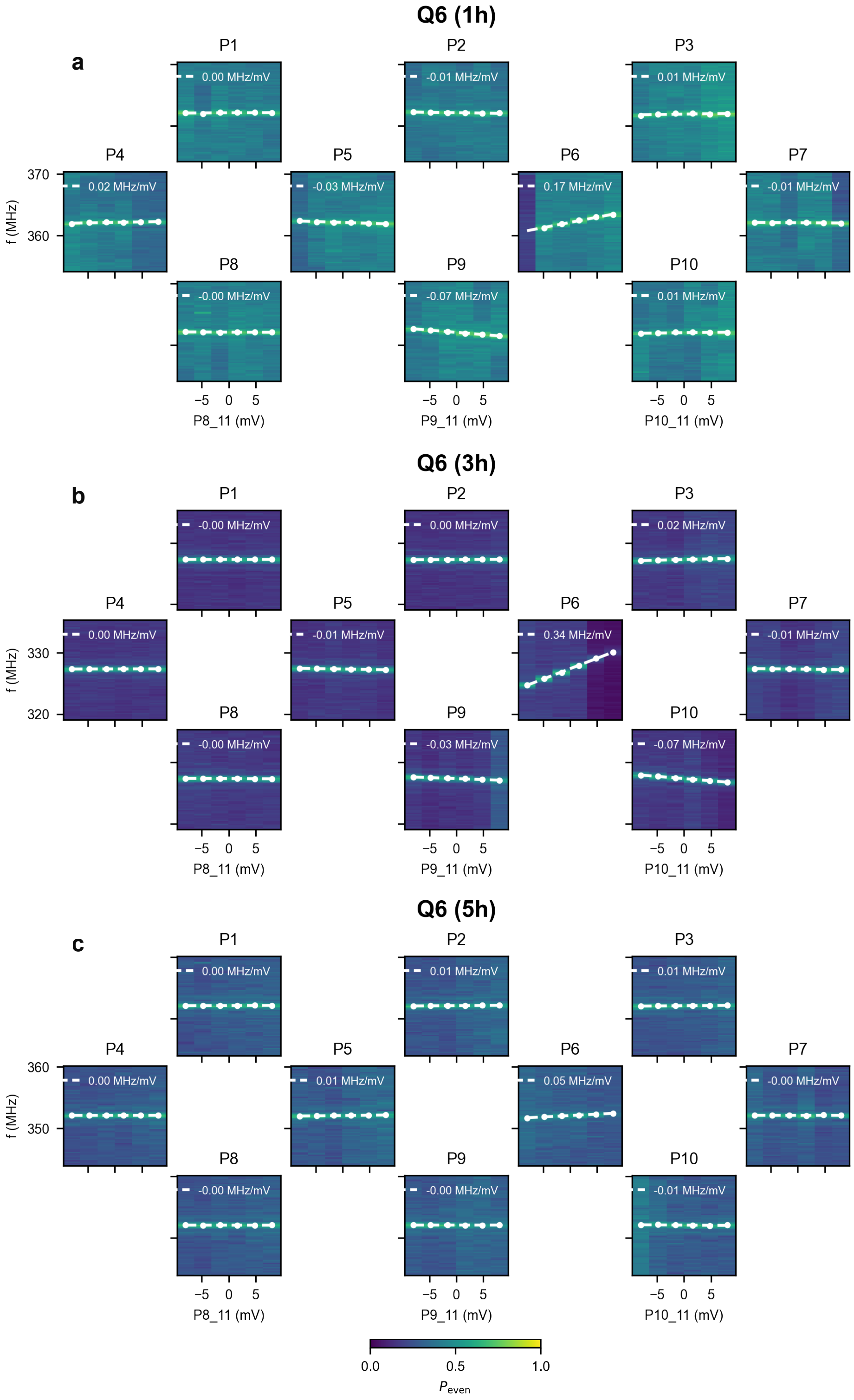

Supplementary Figure S13. **LSES contribution of plungers for Q6 with 1, 3, and 5 hole occupation.**

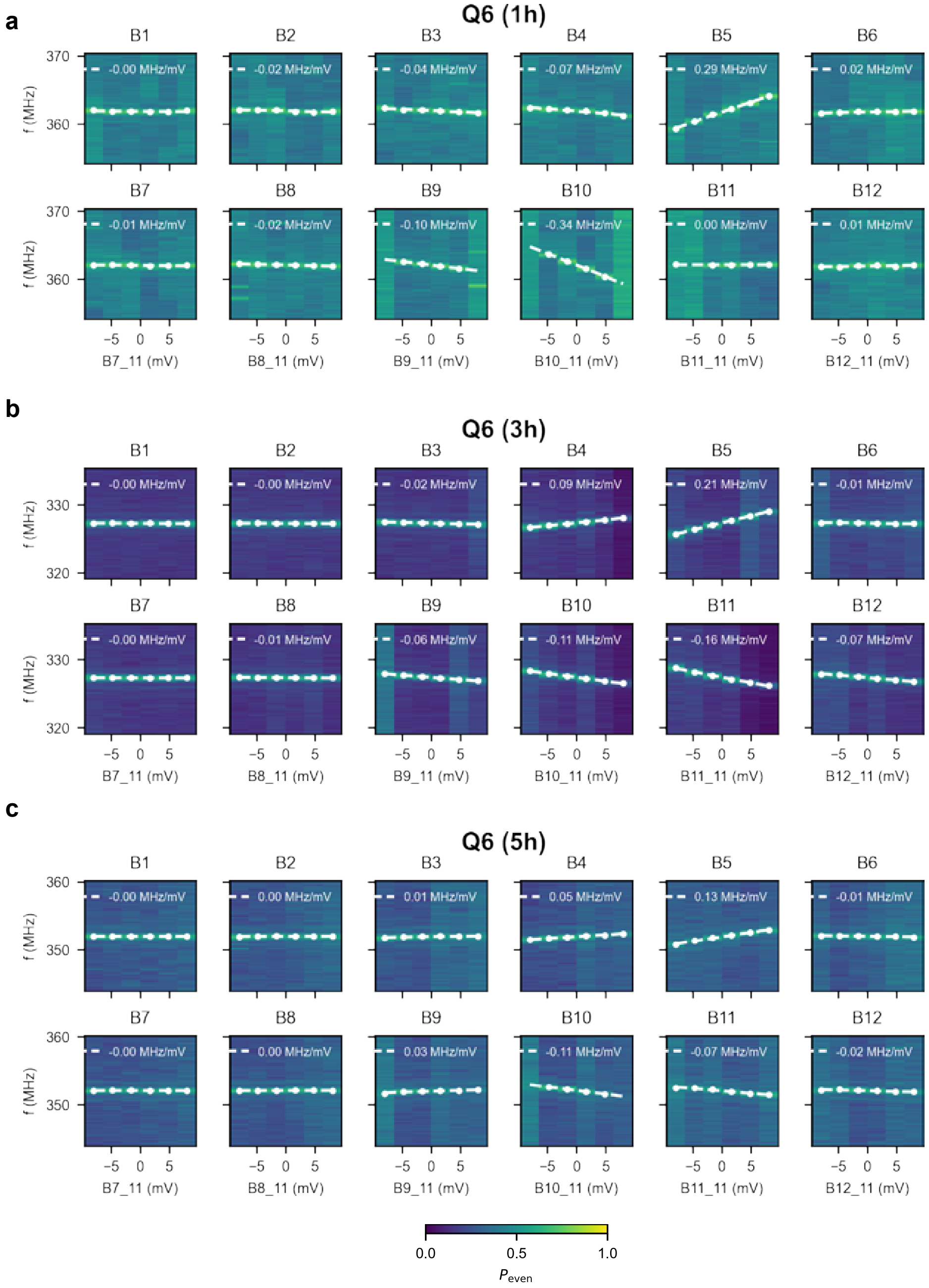

Supplementary Figure S14. **LSES contribution of barriers for Q6 with 1, 3, and 5 hole occupation.**

Suppl. Note 10. DRIVING EFFICIENCY EXTRACTION

To extract the EDSR driving efficiency, Rabi measurements are performed as a function of drive amplitude. Next a fast-Fourier transform is applied to the raw data, and then fitted. In Suppl. Figs. **S15-S18** examples of the data and the corresponding linear fits are plotted. The slope determines the driving efficiency.

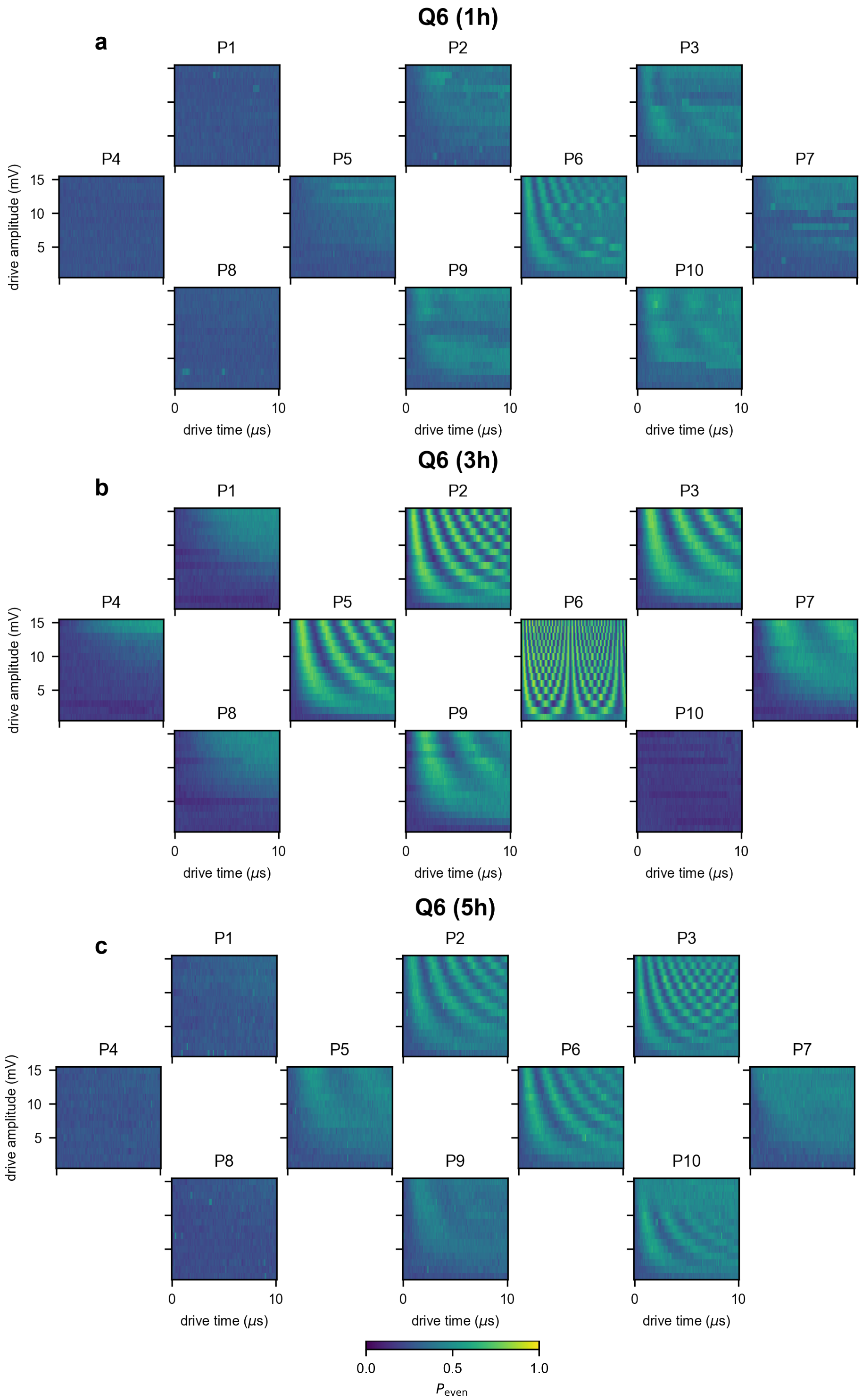

Supplementary Figure S15. **EDSR driving of plungers for Q6 with 1, 3, and 5 hole occupation.**

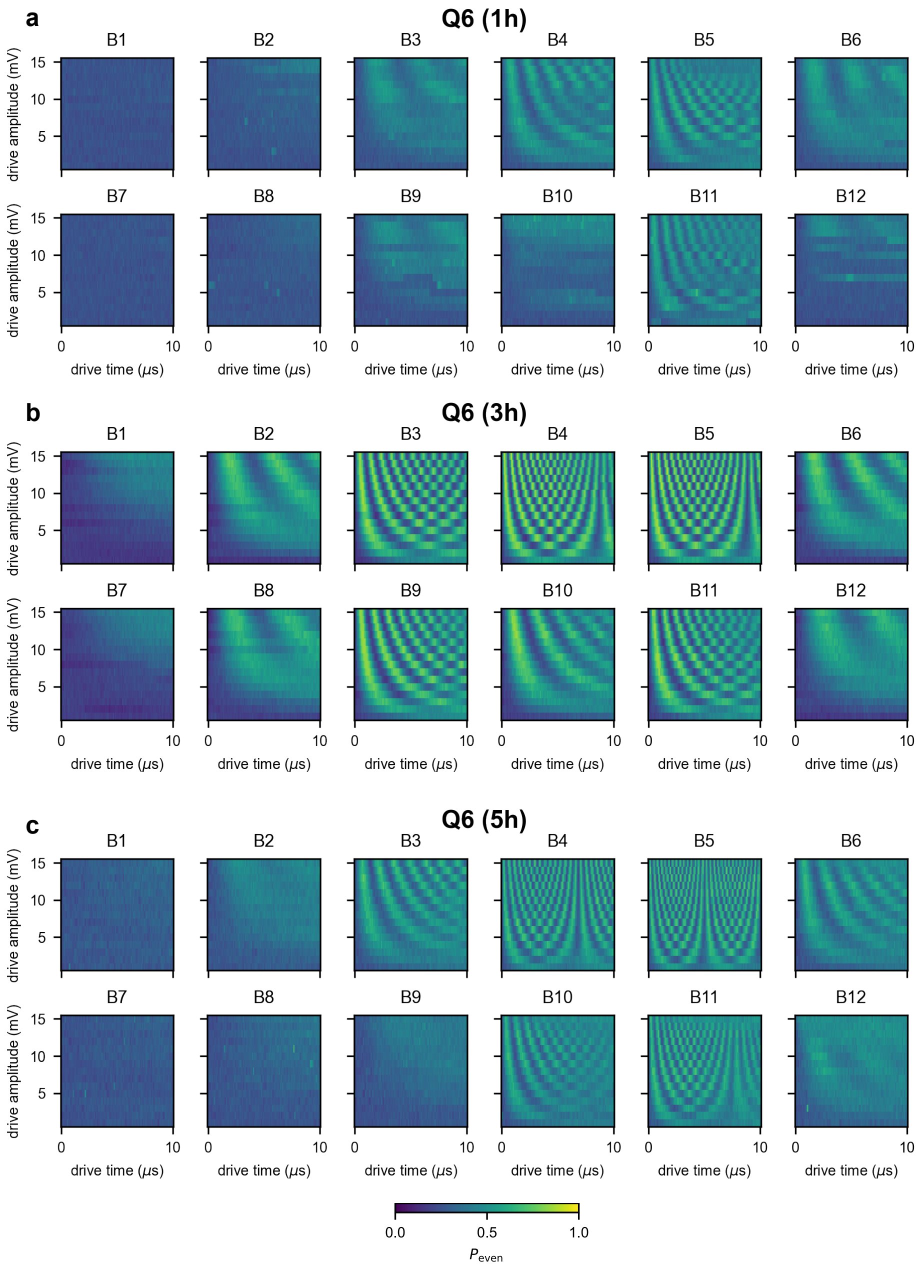

Supplementary Figure S16. **EDSR driving of barriers for Q6 with 1, 3, and 5 hole occupation.**

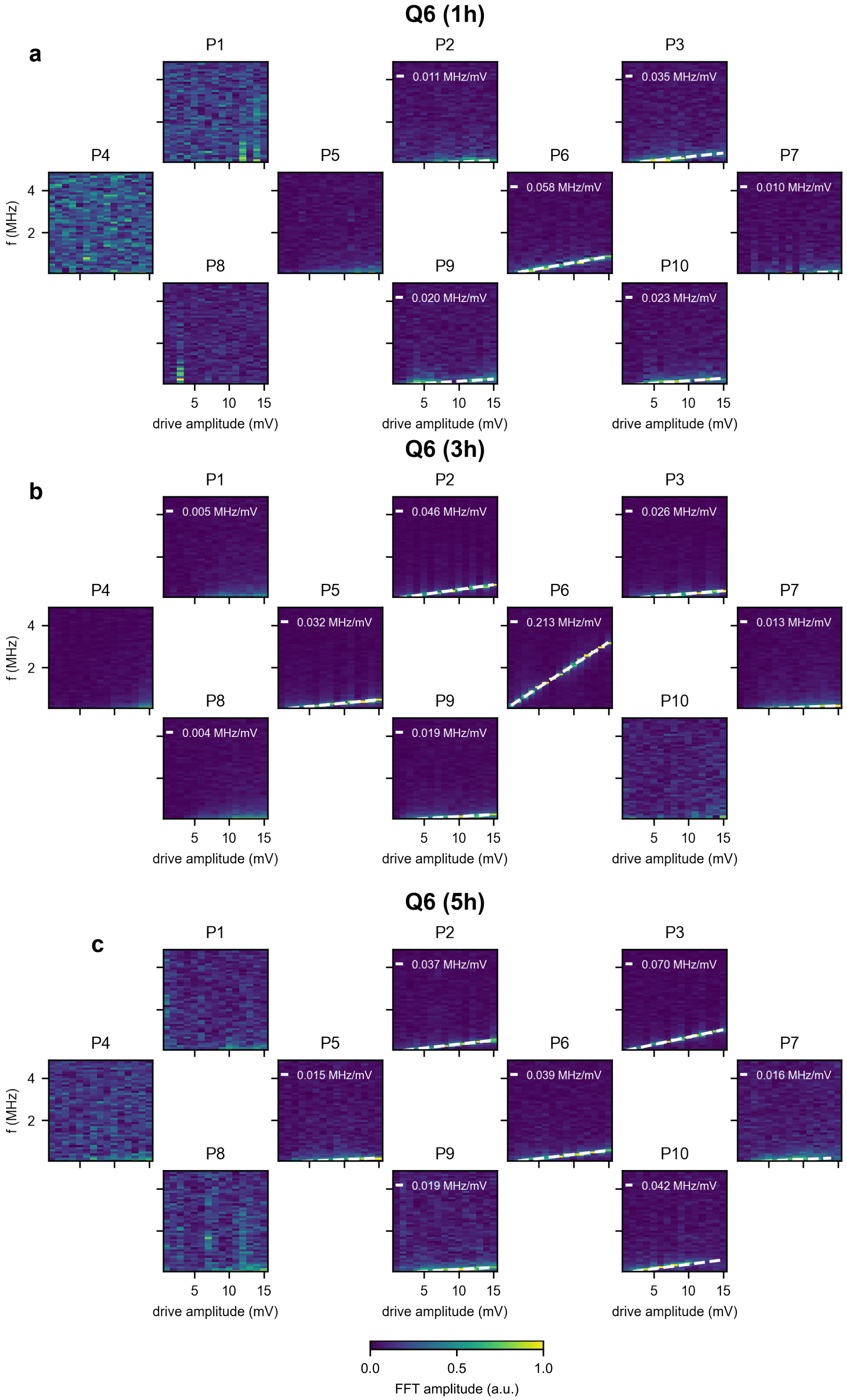

Supplementary Figure S17. FFT and linear fit of EDSR driving of plungers for Q6 with 1, 3, and 5 hole occupation.

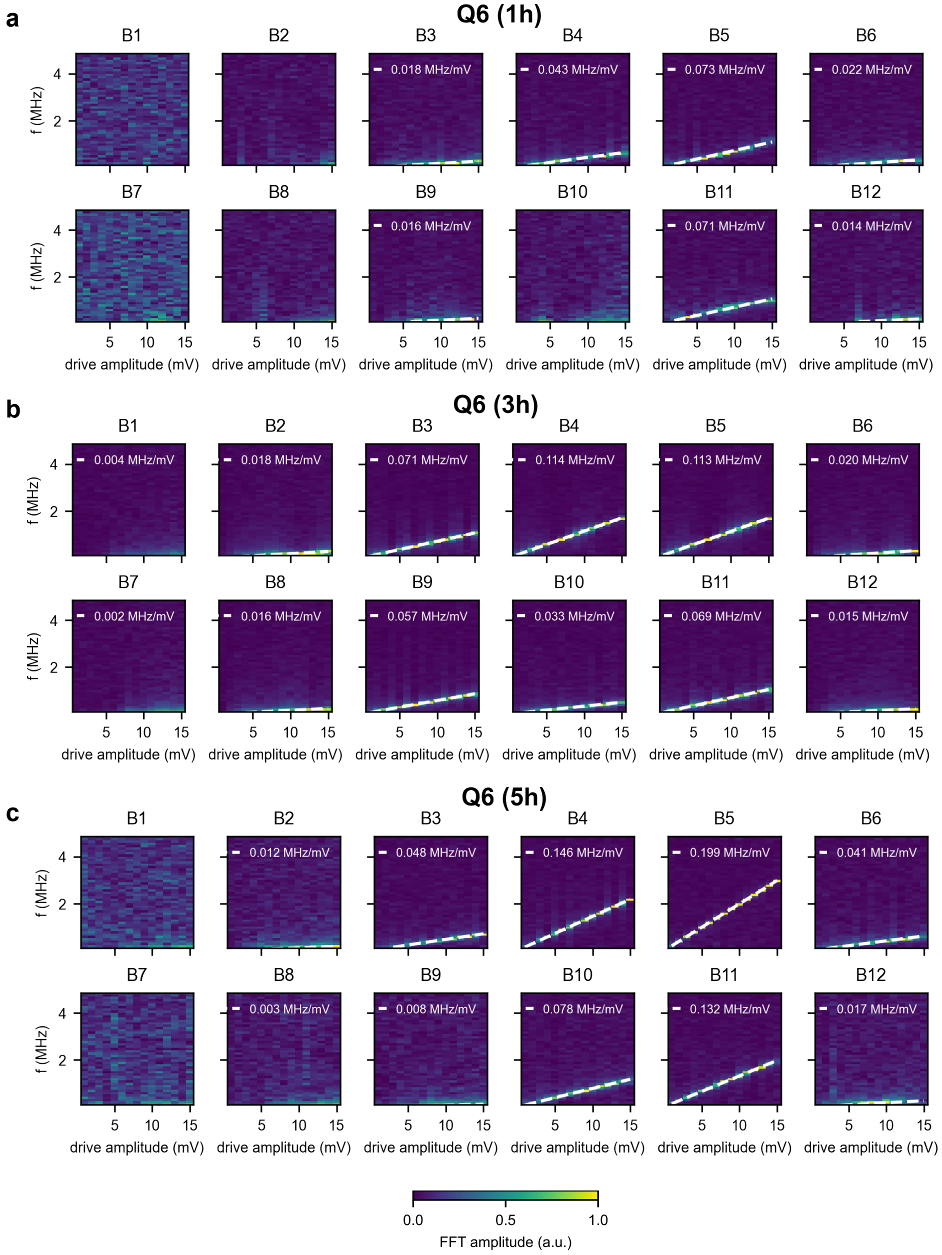

Supplementary Figure S18. **FFT and linear fit of EDSR driving of barriers for Q6 with 1, 3, and 5 hole occupation.**

Suppl. Note 11. PHYSICAL DISTANCE FROM GATES TO QUBITS

To categorise the driving efficiency of all gates to all qubits, we calculate the physical distance in the device plane from the expected qubit position at the centre of its top plunger, to any of the 22 gates. Afterwards, we rank each physical distance for plunger and barrier gates independent from each other, and assign it an integer number n corresponding to the n-th nearest qubit-to-gate distance. We use these assigned ranks to calculate an average value for the n-th nearest qubit for a plunger and barrier gate drive as shown in Figure 4 of the main text.

Supplementary Table S7. Physical distance (rounded to the second digit) from each gate to each qubit in μm

	**P1**	**P2**	**P3**	**P4**	**P5**	**P6**	**P7**	**P8**	**P9**	**P10**	**B1**	**B2**	**B3**	**B4**	**B5**	**B6**	**B7**	**B8**	**B9**	**B10**	**B11**	**B12**
**Q1**	0.00	0.28	0.55	0.20	0.20	0.44	0.70	0.28	0.39	0.62	0.10	0.10	0.22	0.35	0.49	0.62	0.22	0.22	0.29	0.40	0.53	0.65
**Q2**	0.28	0.00	0.28	0.44	0.20	0.20	0.44	0.39	0.28	0.39	0.35	0.22	0.10	0.10	0.22	0.35	0.40	0.29	0.22	0.22	0.29	0.40
**Q3**	0.55	0.28	0.00	0.70	0.44	0.20	0.20	0.62	0.39	0.28	0.62	0.49	0.35	0.22	0.10	0.10	0.65	0.53	0.40	0.29	0.22	0.22
**Q4**	0.20	0.44	0.70	0.00	0.28	0.55	0.83	0.20	0.44	0.70	0.10	0.22	0.35	0.49	0.62	0.76	0.10	0.22	0.35	0.49	0.62	0.76
**Q5**	0.20	0.20	0.44	0.28	0.00	0.28	0.55	0.20	0.20	0.44	0.22	0.10	0.10	0.22	0.35	0.49	0.22	0.10	0.10	0.22	0.35	0.49
**Q6**	0.44	0.20	0.20	0.55	0.28	0.00	0.28	0.44	0.20	0.20	0.49	0.35	0.22	0.10	0.10	0.22	0.49	0.35	0.22	0.10	0.10	0.22
**Q7**	0.70	0.44	0.20	0.83	0.55	0.28	0.00	0.70	0.44	0.20	0.76	0.62	0.49	0.35	0.22	0.10	0.76	0.62	0.49	0.35	0.22	0.10
**Q8**	0.28	0.39	0.62	0.20	0.20	0.44	0.70	0.00	0.28	0.55	0.22	0.22	0.29	0.40	0.53	0.65	0.10	0.10	0.22	0.35	0.49	0.62
**Q9**	0.39	0.28	0.39	0.44	0.20	0.20	0.44	0.28	0.00	0.28	0.40	0.29	0.22	0.22	0.29	0.40	0.35	0.22	0.10	0.10	0.22	0.35
**Q10**	0.62	0.39	0.28	0.70	0.44	0.20	0.20	0.55	0.28	0.00	0.65	0.53	0.40	0.29	0.22	0.22	0.62	0.49	0.35	0.22	0.10	0.10

Supplementary Table S8. Ranked physical distance determining the n-th nearest neighbours

	**P1**	**P2**	**P3**	**P4**	**P5**	**P6**	**P7**	**P8**	**P9**	**P10**	**B1**	**B2**	**B3**	**B4**	**B5**	**B6**	**B7**	**B8**	**B9**	**B10**	**B11**	**B12**
**Q1**	1	3	6	2	2	5	8	3	4	7	1	1	2	4	6	8	2	2	3	5	7	9
**Q2**	3	1	3	5	2	2	5	4	3	4	4	2	1	1	2	4	5	3	2	2	3	5
**Q3**	6	3	1	8	5	2	2	7	4	3	8	6	4	2	1	1	9	7	5	3	2	2
**Q4**	2	5	8	1	3	6	9	2	5	8	1	2	4	6	8	10	1	2	4	6	8	10
**Q5**	2	2	5	3	1	3	6	2	2	5	2	1	1	2	4	6	2	1	1	2	4	6
**Q6**	5	2	2	6	3	1	3	5	2	2	6	4	2	1	1	2	6	4	2	1	1	2
**Q7**	8	5	2	9	6	3	1	8	5	2	10	8	6	4	2	1	10	8	6	4	2	1
**Q8**	3	4	7	2	2	5	8	1	3	6	2	2	3	5	7	9	1	1	2	4	6	8
**Q9**	4	3	4	5	2	2	5	3	1	3	5	3	2	2	3	5	4	2	1	1	2	4
**Q10**	7	4	3	8	5	2	2	6	3	1	9	7	5	3	2	2	8	6	4	2	1	1

Suppl. Note 12. SPATIAL LOCALITY OF THE ELECTRIC FIELD AND QUBIT DRIVE

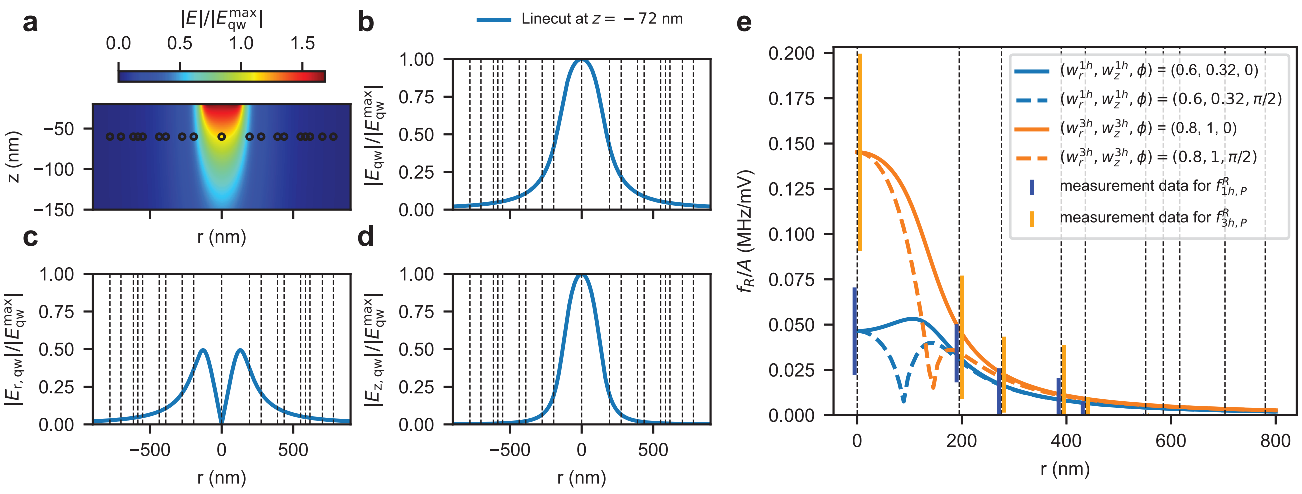

Supplementary Figure S19. **a.** Electric field of a circular disk with diameter 130 nm assuming electric field propagation in vacuum. The black dots indicate the positions of qubits. **b-c.** normalised electric field taken along $z = -72\,\text{nm}$ for E, E_r, and E_z respectively. The dashed vertical lines indicate qubit positions. The red dashed line is a fit of the electric field simulation data to extract a decay exponent of the electric field. **e.** Rabi drive efficiency as a function of spatial distance. The displayed data including the represented standard deviation is the same as in Fig. 4b and d of the main manuscript. The continuous and dashed lines indicate weighted drive contribution estimates of electric field with constructive and deconstructive interference of the r-drive and the z-drive respectively.

We can make a basic electrostatic estimation of the electric field coming from a plunger gate by approximating it as a charged circular disk. The value of the charge density is not important here, since we are only interested in the relative change of the electric field as a function of the vertical distance z and the radial distance r. We assume electric field propagation in vacuum and evaluate the electric field along r and z by integrating the contribution of each point of the charged disk using Coulomb law. We also assume the qubit site to be a single point in space. In Figure **S19**a you can see the total electric field at each point for z ranging from -20 nm to -150 nm and r ranging from -800 nm to $+800$ nm, normalised to the maximum of the total electric field E_qw^max at $z = -72$ nm. The positions of qubit sites relative to the plunger gate positions are indicated by the black dots. In Figure **S19**b a linecut of the total electric field E_qw has been taken at $z = -72$ nm, which corresponds to the distance of the plunger gate layer to the quantum well. Hence, we denote the linecut with the subscript qw. The dashed vertical lines indicate qubit sites relative to the plunger gate. In Figure **S19**c and d we separate the electric field contribution into a radial (E_r) and horizontal (E_z) part. We can see that E_z falls of substantially even towards the nearest neighbour. In contrast the radial electric field starts to dominate for all qubit sites of the nearest neighbour and beyond.

To connect the electric field decay with the observed locality of the qubit drive, we assume that we can fully separate the qubit drive coming from E_r and E_z. We note that in a more accurate picture, the electric field of the qubit is non-separable [S8]. Since the vertical and horizontal driving mechanism differ, we can assign relative weights to these driving mechanisms (denoted w_r^1h, w_z^1h, w_r^3h, and w_z^3h), such that the experimentally observed decay in driving efficiency matches

$$\frac{f_\text{Rabi}}{A} \propto \sqrt{|(w_r E_r)^2 + (w_z E_z e^{i\phi})^2|} \tag{23}$$

where ϕ is the phase difference between the radial and horizontal drive. Such an assignment of the weights is shown in Figure **S19**e, where $w_z^{3h} = 1$ and all other weights are relative to that. The result from assigning these weights can be seen in Figure **S19**e, where the proportionality is compared with the measurement data of $f^R_{1h,P}$ and $f^R_{3h,P}$, which is also displayed in Fig. 4b and d of the main manuscript. It shows very good agreement with the experimental data. Interestingly, the assigned phase difference does not make a big difference since heavy constructive and deconstructive interference is only happening before the electric field hits the first neighbouring qubit site. The assigned weights also align well with the the FCI simulations. We know from Figure 3 of the main manuscript that barrier drive, which has a significant E_r component, does not change as much between 1-hole and 3-hole occupation. This is in contrast

to the plunger drive, which has exclusively an E_z component. These observations are reflected in the weights, where w_r^{1h} and w_r^{3h} are similar but w_z^{1h} and w_z^{3h} change significantly.

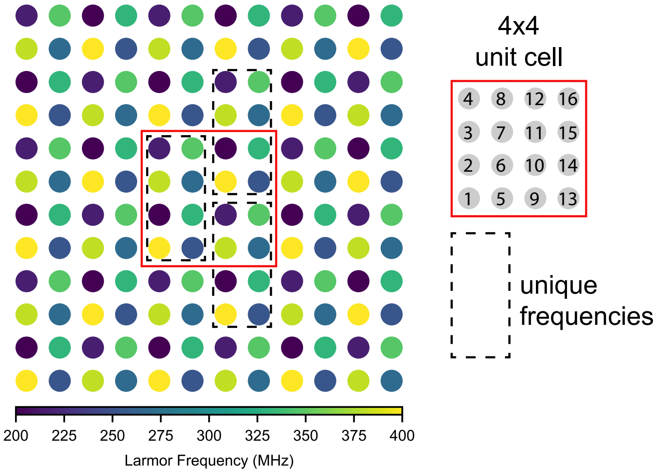

Supplementary Figure S20. Eight unique frequencies that can make up an infinite dense qubit array. The frequencies are selected within a 4x4 unit cell.

Suppl. Note 13. FREQUENCY CROSS-TALK

The cross-talk analysis in the main text is focused on achieving locality in qubit driving independent of frequency detuning between neighbouring qubits. Specifically, we analysed crosstalk under the worst-case scenario where all qubits share the same resonance frequency. This sets the minimum distance at which no two qubits should operate at the same frequency.

If assuming that we can reliably target Larmor frequencies, it becomes possible to operate a quasi-periodic two-dimensional qubit array composed of repeating tiles QX, each requiring only eight distinct Larmor frequencies. We note that due to its electrical tunability these frequencies can be tuned around the given target value. An example of such a frequency mapping is shown in Fig. **S20**, where Larmor frequencies between 200 and 400 MHz have been assigned across the array. The nearest-neighbour resonance frequency differences range from 62 MHz between QX6 and QX7 to 200 MHz between QX11 and QX12.

To estimate the local qubit-to-qubit crosstalk $\chi_{i,j}^{\mathrm{local}}$, we combine the measured spatial decay of drive strength (i.e. its dependence on nearest-neighbour distance) with a frequency-dependent suppression factor coming from the magnitude-squared of the complex valued spectral density of the pulse shape. Specifically, we approximate the global crosstalk contribution between two qubits using the following expression, which constitutes the worst-case scenario assuming a square pulse envelope:

$$\chi_{i,j}^{\mathrm{global}} = \frac{f_{\mathrm{rabi}}^2}{f_{\mathrm{rabi}}^2 + (f_{Qi} - f_{Qj})^2} \tag{24}$$

where f_{rabi} is the Rabi frequency of the driven qubit. The full local crosstalk $\chi_{i,j}^{\mathrm{local}}$ is then obtained by weighting this frequency response with the spatially dependent drive strength extracted from the nearest-neighbour dataset (Fig. 4d of the main text):

$$\chi_{i,j}^{local} = A(d_{i,j}) \cdot \chi_{i,j}^{global} \tag{25}$$

where $A(d_{i,j})$ is the experimentally determined rotation of the residual drive at the location of qubit j when driving qubit i, as a function of their distance $d_{i,j}$. This approach incorporates both spatial and spectral selectivity, and provides a quantitative estimate of crosstalk for arbitrary frequency allocations. We note that for higher precision estimates it becomes important to accurately resolve the tail of the electric field shown in Figure **S19**e and the effect of screening. Here, we have solely considered the measurement data, which were only sensitive enough up to $\sim 0.08\,\mathrm{MHz/mV}$, which corresponds to a horizontal distance of $< 500\,\mathrm{nm}$. Table **S9** summarises both the maximum $\max\left(\chi_{i,j}^{local}\right)$ and the total $\sum \chi_{i,j}^{local}$ crosstalk per qubit. The maximum values range from 10^{-5} to 10^{-4}, and the total crosstalk from 10^{-4} to 10^{-3}. Pulse shaping can further suppress these values by many orders of magnitude [S21–S24].

Usually it is beneficial to have a Δg between neighbouring qubits as large as possible, as long as the Larmor frequencies remain large enough to avoid triggering multi-photon resonances [S25, S26] and breaking the rotating wave approximation [S27]. However, commonly one has to adapt to other operation and design constraints that only

Supplementary Table S9. Assigned Larmor frequencies and experienced crosstalk.

subnumber i	f_{Larmor} (MHz)	$\max_j(\chi^{\mathrm{local}}_{i,j})(10^{-3})$	$\sum_j \chi^{\mathrm{local}}_{i,j}(10^{-3})$
1	400	0.08	0.52
2	200	0.08	0.52
3	383	0.09	0.66
4	217	0.09	0.66
5	253	0.09	0.76
6	331	0.09	0.70
7	269	0.09	0.70
8	347	0.09	0.76
9	383	0.09	0.66
10	217	0.09	0.66
11	400	0.08	0.52
12	200	0.08	0.52
13	269	0.09	0.70
14	347	0.09	0.76
15	253	0.09	0.76
16	331	0.09	0.70

allow Larmor frequencies within a certain window, e.g. 200 - 400 MHz to be able to drive them with state-of-the-art arbitrary waveform generators (considering convenience and economics).

Suppl. Note 14. SCALABILITY CONSIDERATIONS AND VARIABILITY

While intrinsic spin-orbit coupling is a convenient tool for spin qubit manipulation, it also opens up significant challenges when moving towards the operation of large qubit arrays. This manifests itself in a certain variability of the g-tensor across all qubit sites, which largely determines qubit dynamics. It is critical on how to deal with such variability. Generally, we envision to have the largest uniformity as possible and rather engineer differences in the g-tensor (if desired) instead of relying on the natural variability. The engineering of the g-tensor can happen through confinement potentials in different shape and sizes, either by hardcoding the shape and size into the plunger gate design or by utilising additional side gates that can tune the shape and size dynamically, similar to Ref. [S17]. On a larger scale (µm-scale) one has to cope with the strain differences naturally arising from the SiGe strain relaxed buffers (SRBs) [S28, S29]. These strain differences vary continuously along the [110] and [$\bar{1}$10] crystal directions with a periodic pattern of $1-5\,\mu\mathrm{m}$ [S29, S30]. This can be dealt with by tuning it back to a constant base level across the full device, or embracing a periodic pattern that allows g-tensor differences needed for manipulation schemes such as hopping-based qubit operations [S31]. The level of uniformity needed and the required ability to dynamically control the g-tensor highly depend on the dedicated encoding and architecture. For a sparse qubit array operated through EDSR one could aim for maximum uniformity with a magnetic field slightly out-of-plane. For hopping spin architectures or dense qubit arrays operated through ESDR, it may be necessary to engineer g-tensor differences.

Suppl. Note 15. ERROR CORRECTION

Error correction codes are critical for achieving large-scale quantum computation [S32]. The distance-2 surface code requires four data qubits, three ancilla qubits, and additional three reference qubits to read out the ancilla qubits (if relying on PSB). If aiming to perform such a code on a similar device as investigated in this study, the data qubits could be placed on quantum dot sites 1, 3, 8 and 10. The three ancilla qubits can be placed on qubit sites 4, 7, and 9. The ancilla qubit in quantum dot 9 can reach all 4 data qubits by means of shuttling to quantum dot 5 and 6. The other two ancilla qubits are direct neighbours of the two qubits that they are supposed to check. The read-out of the ancilla qubits would require an additional reference qubit for each ancilla qubit. The reference qubit for the centre ancilla qubit can be placed in quantum dot 2. The reference qubits for the other two ancilla qubits would require an additional quantum dot on each side (left and right).

We note that in a proof-of-concept experiment one would also want to read-out the four data qubits independently to confirm that the stabilisers returns the expected eigenvalue. This requires either the implementation of even more

reference qubits, or (preferably) sequential read-out with the same reference qubit. However, the latter works optimal when $T_{\mathrm{measure}} << T_2^*$, which is not routinely obtained yet in state-of-the-art spin qubit experiments.

[S1] X. Xue, T. Watson, J. Helsen, D. Ward, D. Savage, M. Lagally, S. Coppersmith, M. Eriksson, S. Wehner, and L. Vandersypen, Benchmarking Gate Fidelities in a Si/SiGe Two-Qubit Device, Physical Review X **9**, 021011 (2019).

[S2] J. J. Wallman, M. Barnhill, and J. Emerson, Robust characterization of leakage errors, New Journal of Physics **18**, 043021 (2016).

[S3] E. Nielsen, J. K. Gamble, K. Rudinger, T. Scholten, K. Young, and R. Blume-Kohout, Gate Set Tomography, Quantum **5**, 557 (2021).

[S4] A. S. Rao, D. Buterakos, B. van Straaten, V. John, C. X. Yu, S. D. Oosterhout, L. Stehouwer, G. Scappucci, M. Veldhorst, F. Borsoi, and J. P. Zwolak, Modular Autonomous Virtualization System for Two-Dimensional Semiconductor Quantum Dot Arrays, Physical Review X **15**, 021034 (2025).

[S5] J. M. Luttinger and W. Kohn, Motion of Electrons and Holes in Perturbed Periodic Fields, Physical Review **97**, 869 (1955).

[S6] M. Willatzen and L. C. Lew Yan Voon, *The k p Method: Electronic Properties of Semiconductors* (Springer Berlin Heidelberg, Berlin, Heidelberg, 2009) pp. 1–445.

[S7] B. Venitucci, L. Bourdet, D. Pouzada, and Y. M. Niquet, Electrical manipulation of semiconductor spin qubits within the g -matrix formalism, Physical Review B **98**, 155319 (2018).

[S8] B. Martinez, J. C. Abadillo-Uriel, E. A. Rodríguez-Mena, and Y.-M. Niquet, Hole spin manipulation in inhomogeneous and nonseparable electric fields, Physical Review B **106**, 235426 (2022).

[S9] L. Mauro, E. A. Rodríguez-Mena, M. Bassi, V. Schmitt, and Y.-M. Niquet, Geometry of the dephasing sweet spots of spin-orbit qubits, Physical Review B **109**, 155406 (2024).

[S10] J. C. Abadillo-Uriel, B. Martinez, M. Filippone, and Y. M. Niquet, Two-body Wigner molecularization in asymmetric quantum dot spin qubits, Physical Review B **104**, 195305 (2021).

[S11] J. C. Abadillo-Uriel, E. A. Rodríguez-Mena, B. Martinez, and Y.-M. Niquet, Hole-Spin Driving by Strain-Induced Spin-Orbit Interactions, Physical Review Letters **131**, 097002 (2023).

[S12] A. Abragam and B. Bleaney, *Electron Paramagnetic Resonance of Transition Ions* (Presses Universitaires de France, 1971).

[S13] S. Bosco, M. Benito, C. Adelsberger, and D. Loss, Squeezed hole spin qubits in Ge quantum dots with ultrafast gates at low power, Physical Review B **104**, 115425 (2021).

[S14] B. Martinez, S. de Franceschi, and Y.-M. Niquet, Mitigating variability in epitaxial-heterostructure-based spin-qubit devices by optimizing gate layout, Physical Review Applied **22**, 024030 (2024).

[S15] G. Burkard, T. D. Ladd, A. Pan, J. M. Nichol, and J. R. Petta, Semiconductor spin qubits, Reviews of Modern Physics **95**, 025003 (2023).

[S16] N. Ares, V. N. Golovach, G. Katsaros, M. Stoffel, F. Fournel, L. I. Glazman, O. G. Schmidt, and S. De Franceschi, Nature of tunable hole g factors in quantum dots, Physical Review Letters **110**, 046602 (2013).

[S17] M. Rimbach-Russ, V. John, B. van Straaten, and S. Bosco, A spinless spin qubit, arXiv (2024).

[S18] D. V. Bulaev and D. Loss, Electric Dipole Spin Resonance for Heavy Holes in Quantum Dots, Physical Review Letters **98**, 097202 (2007).

[S19] L. A. Terrazos, E. Marcellina, Z. Wang, S. N. Coppersmith, M. Friesen, A. R. Hamilton, X. Hu, B. Koiller, A. L. Saraiva, D. Culcer, and R. B. Capaz, Theory of hole-spin qubits in strained germanium quantum dots, Physical Review B **103**, 125201 (2021).

[S20] M. J. Rodríguez, E. A. Rodríguez-Mena, A. F. Kalo, and Y.-M. Niquet, Dressed basis sets for the modeling of exchange interactions in double quantum dots, arXiv (2025).

[S21] L. S. Theis, F. Motzoi, S. Machnes, and F. K. Wilhelm, Counteracting systems of diabaticities using DRAG controls: The status after 10 years(a), Europhysics Letters **123**, 60001 (2018).

[S22] I. Heinz and G. Burkard, Crosstalk analysis for single-qubit and two-qubit gates in spin qubit arrays, Physical Review B **104**, 045420 (2021).

[S23] M. Rimbach-Russ, S. G. Philips, X. Xue, and L. M. Vandersypen, Simple framework for systematic high-fidelity gate operations, Quantum Science and Technology **8**, 045025 (2023).

[S24] Y.-H. Wu, L. C. Camenzind, P. Bütler, I. K. Jin, A. Noiri, K. Takeda, T. Nakajima, T. Kobayashi, G. Scappucci, H.-S. Goan, and S. Tarucha, Simultaneous High-Fidelity Single-Qubit Gates in a Spin Qubit Array, arXiv (2025).

[S25] V. John, F. Borsoi, Z. György, C.-A. Wang, G. Széchenyi, F. van Riggelen-Doelman, W. I. Lawrie, N. W. Hendrickx, A. Sammak, G. Scappucci, A. Pályi, and M. Veldhorst, Bichromatic Rabi Control of Semiconductor Qubits, Physical Review Letters **132**, 067001 (2024).

[S26] Z. György, A. Pályi, and G. Széchenyi, Electrically driven spin resonance with bichromatic driving, Physical Review B **106**, 155412 (2022).

[S27] D. A. Rower, L. Ding, H. Zhang, M. Hays, J. An, P. M. Harrington, I. T. Rosen, J. M. Gertler, T. M. Hazard, B. M. Niedzielski, M. E. Schwartz, S. Gustavsson, K. Serniak, J. A. Grover, and W. D. Oliver, Suppressing Counter-Rotating Errors for Fast Single-Qubit Gates with Fluxonium, PRX Quantum **5**, 040342 (2024).

[S28] M. Lodari, N. W. Hendrickx, W. I. L. Lawrie, T.-K. Hsiao, L. M. K. Vandersypen, A. Sammak, M. Veldhorst, and G. Scappucci, Low percolation density and charge noise with holes in germanium, Materials for Quantum Technology **1**, 011002 (2021).

[S29] L. E. A. Stehouwer, A. Tosato, D. Degli Esposti, D. Costa, M. Veldhorst, A. Sammak, and G. Scappucci, Germanium wafers for strained quantum wells with low disorder, Applied Physics Letters **123**, 92101 (2023).

[S30] C. Corley-Wiciak, C. Richter, M. H. Zoellner, I. Zaitsev, C. L. Manganelli, E. Zatterin, T. U. Schülli, A. A. Corley-Wiciak, J. Katzer, F. Reichmann, W. M. Klesse, N. W. Hendrickx, A. Sammak, M. Veldhorst, G. Scappucci, M. Virgilio, and G. Capellini, Nanoscale Mapping of the 3D Strain Tensor in a Germanium Quantum Well Hosting a Functional Spin Qubit Device, ACS Applied Materials & Interfaces **15**, 3119 (2023).

[S31] C.-A. Wang, V. John, H. Tidjani, C. X. Yu, A. S. Ivlev, C. Déprez, F. van Riggelen-Doelman, B. D. Woods, N. W. Hendrickx, W. I. L. Lawrie, L. E. A. Stehouwer, S. D. Oosterhout, A. Sammak, M. Friesen, G. Scappucci, S. L. de Snoo, M. Rimbach-Russ, F. Borsoi, and M. Veldhorst, Operating semiconductor quantum processors with hopping spins, Science **385**, 447 (2024).

[S32] A. G. Fowler, M. Mariantoni, J. M. Martinis, and A. N. Cleland, Surface codes: Towards practical large-scale quantum computation, Physical Review A **86**, 032324 (2012).
